# A Holistic Data-Driven Approach to Synthesis Predictions of Colloidal Nanocrystal Shapes

**DOI:** 10.1021/jacs.4c17283

**Published:** 2025-02-07

**Authors:** Ludovic Zaza, Bojana Ranković, Philippe Schwaller, Raffaella Buonsanti

**Affiliations:** 1 Laboratory of Nanochemistry for Energy (LNCE), Department of Chemical Sciences and Engineering, École Polytechnique Fédérale de Lausanne, Sion CH-1950, Switzerland; 2 Laboratory of Artificial Chemical Intelligence (LIAC), Department of Chemical Sciences and Engineering, École Polytechnique Fédérale de Lausanne, Lausanne CH-1015, Switzerland

## Abstract

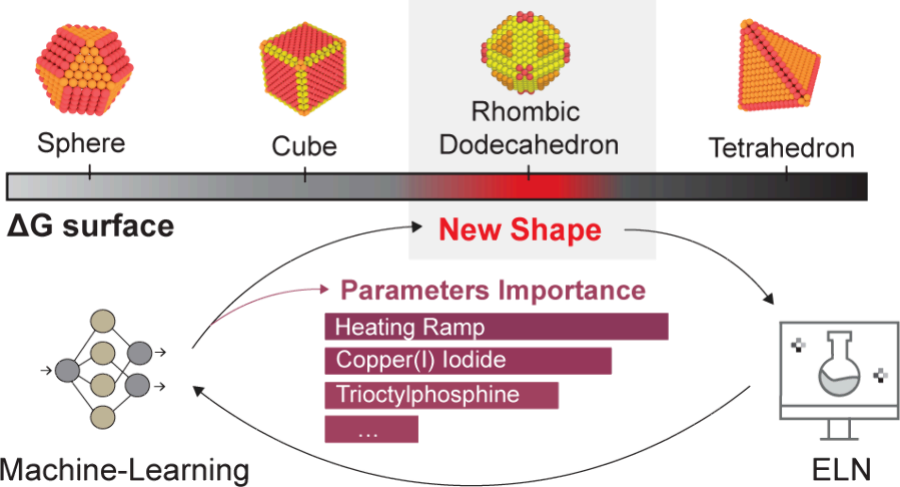

The ability to precisely design colloidal nanocrystals (NCs) has far-reaching implications in optoelectronics, catalysis, biomedicine, and beyond. Achieving such control is generally based on a trial-and-error approach. Data-driven synthesis holds promise to advance both discovery and mechanistic knowledge. Herein, we contribute to advancing the current state of the art in the chemical synthesis of colloidal NCs by proposing a machine-learning toolbox that operates in a low-data regime, yet comprehensive of the most typical parameters relevant for colloidal NC synthesis. The developed toolbox predicts the NC shape given the reaction conditions and proposes reaction conditions given a target NC shape using Cu NCs as the model system. By classifying NC shapes on a continuous energy scale, we synthesize an unreported shape, which is the Cu rhombic dodecahedron. This holistic approach integrates data-driven and computational tools with materials chemistry. Such development is promising to greatly accelerate materials discovery and mechanistic understanding, thus advancing the field of tailored materials with atomic-scale precision tunability.

## 1 Introduction

Well-defined nanocrystals (NCs) are important nanomaterials for a wide range of applications in optoelectronics, catalysis, and biomedicine. The properties of these nanomaterials are uniquely tunable via their size, shape, and bulk composition.^1,2^ Huge progress has been made in the synthesis of NCs, culminating with the 2023 Nobel Prize in chemistry for colloidal quantum dots. Yet, not all colloidal NC materials are currently accessible with the same precision and tunability.

Data-driven and machine-learning (ML) methods have found wide application in organic chemistry for the discovery of chemicals, synthesis optimization, and mechanistic understanding.^3−8^ Challenges exist for the implementation of these methods to materials chemistry due to the increased complexity resulting from the larger compositional space, different synthesis methods (i.e., colloidal methods, solid state chemistry, hydrothermal synthesis), the variety of target properties, and the characterization that comes with those. Yet, recent studies do demonstrate the extension of data-driven methods to material chemistry, mostly in connection with unique high-throughput platforms.^9−11^ Among these, studies on data-driven synthetic optimization of colloidal NCs have generally focused on optical properties or composition/phase control, which are more suitable to continuous monitoring.^11−15^ In parallel to these high-throughput efforts, text mining of literature data has been pursued.^11,16^ However, the data are not always reliable because of the lack of availability and the lack of standardization of data collection and reporting.^17,18^ In this scenario, developing methods that address a low-data regime (i.e., with less than 200 experimental data points) in individual laboratories would be beneficial. Studies operating in this regime remain rare.^12,13^

NC shape control is crucial in many applications because of the relationship between the NC shape, the exposed crystallographic facets, and the properties (e.g., selectivity in heterogeneous catalysis and local field enhancement in plasmonics).^1^ Therefore, shape tuning is important to precisely tailor NC properties and, eventually, to maximize the performance in the application of interest.^1,19^ Typically, synthetic development is based on trial-and-error experiments. The experimental planning relies on expertise and insights from the literature, which help to narrow the design space. However, the number of experiments to reach the target material, when reached, is often extensive and time-consuming (e.g., ∼200 syntheses taking months to 1–2 years to target a new NC shape). Bayesian optimization (BO) methods hold the promise to accelerate this synthetic process by reducing the number of trials via efficiently sampling the design space of all possible experiments.^20−23^ The BO backbone allows for a dynamic search toward the targeted objectives by balancing two important goals: exploring new reactions to improve understanding of the underlying chemical process and focusing on the most promising reactions. However, the application of BO to NC shape control has not been explored so far.

Herein, we propose a holistic approach of data-driven ML for the synthesis of colloidal nanocrystals that operates in a low-data regime, links reaction parameters and the resultant NC shape in a bidirectional manner, and finally predicts the parameters for discovery of new shapes. We chose Cu NCs as a model example because of their shape-dependent catalytic and plasmonic properties^24−26^ and of their underdeveloped chemistry compared to other classes of colloidal NCs.^1,26^ The ML models allow the identification of the main variables in colloidal synthesis that correlate with specific morphological outcomes and, in addition, generate synthetic recipes to target desired NC shapes. Finally, we uniquely introduce a continuous scale for NC shape classification, which enables the models to generate synthetic conditions to target new NC shapes.

## 2 Results and Discussion

### 2.1 Bridging the Gap between NC Synthesis and Machine-Actionable Data

**Figure 1:**
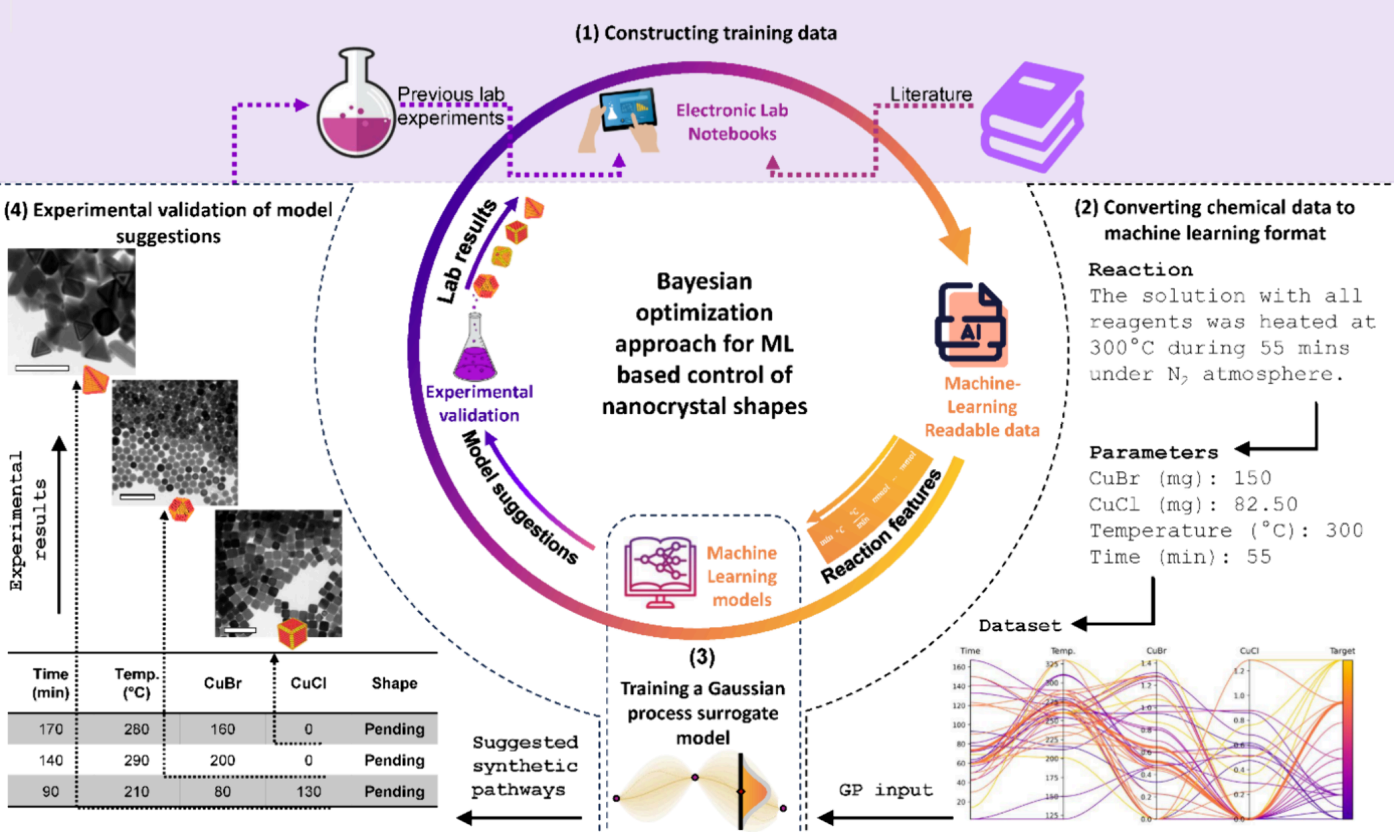
Workflow employed for the synthesis predictions of colloidal NC shapes. (1) Integrating experimental data from the literature and the laboratory within the electronic lab notebook (ELN). (2) Extracting ELN data in a machine-learning readable format. (3) Training a surrogate model that predicts the nanocrystal shapes alongside the associated uncertainties from synthetic variables. The model outputs suggestions for the next experiments to be performed in the laboratory. (4) Experimental validation of the proposed syntheses. After evaluating the syntheses, the results are entered into the ELN and used for the next iteration. The process repeats until reaching the targeted objective. TEM scale bars are 200 nm.

Our workflow consists of a closed loop from the training data in the electronic lab notebook (ELN) to the experimental validation of the models (Figure 1). The ELN plays a pivotal role as a comprehensive record-keeping tool that facilitates data sharing between the laboratory and data scientists.^27,28^ We created a machine-readable database by integrating data from literature and data from conducted experiments in our laboratory into the ELN. We chose to include in the database a number of experimental variables that are as comprehensive as possible of colloidal synthesis (i.e., different precursors, ligands, hot injection, heat up, temperature, time, and heating ramp)^29,30^ to establish a robust design space for optimization. Each experiment recorded in the ELN defines a single input data point for the ML model training. Compiling all information into the ELN resulted in an initial data set of 115 data points used as a base for ML predictive tasks.

Unlike typical machine learning applications that rely on massive data sets, we work with a relatively small number of experimental data points. This data-limited scenario is common in materials synthesis where each data point requires significant time and resources.^21,31,32^ As detailed below, we demonstrate that the obtained data set can be used to gain useful chemical insights and provides a starting point for targeted synthesis optimization using BO methods. BO operates well within the low-data scenarios by relying on surrogate models with well-calibrated uncertainties.^33^ Thus, BO methods emerge as suitable for material synthesis optimization.

### 2.2 Extracting Chemical Insights from NC Synthesis

**Figure 2:**
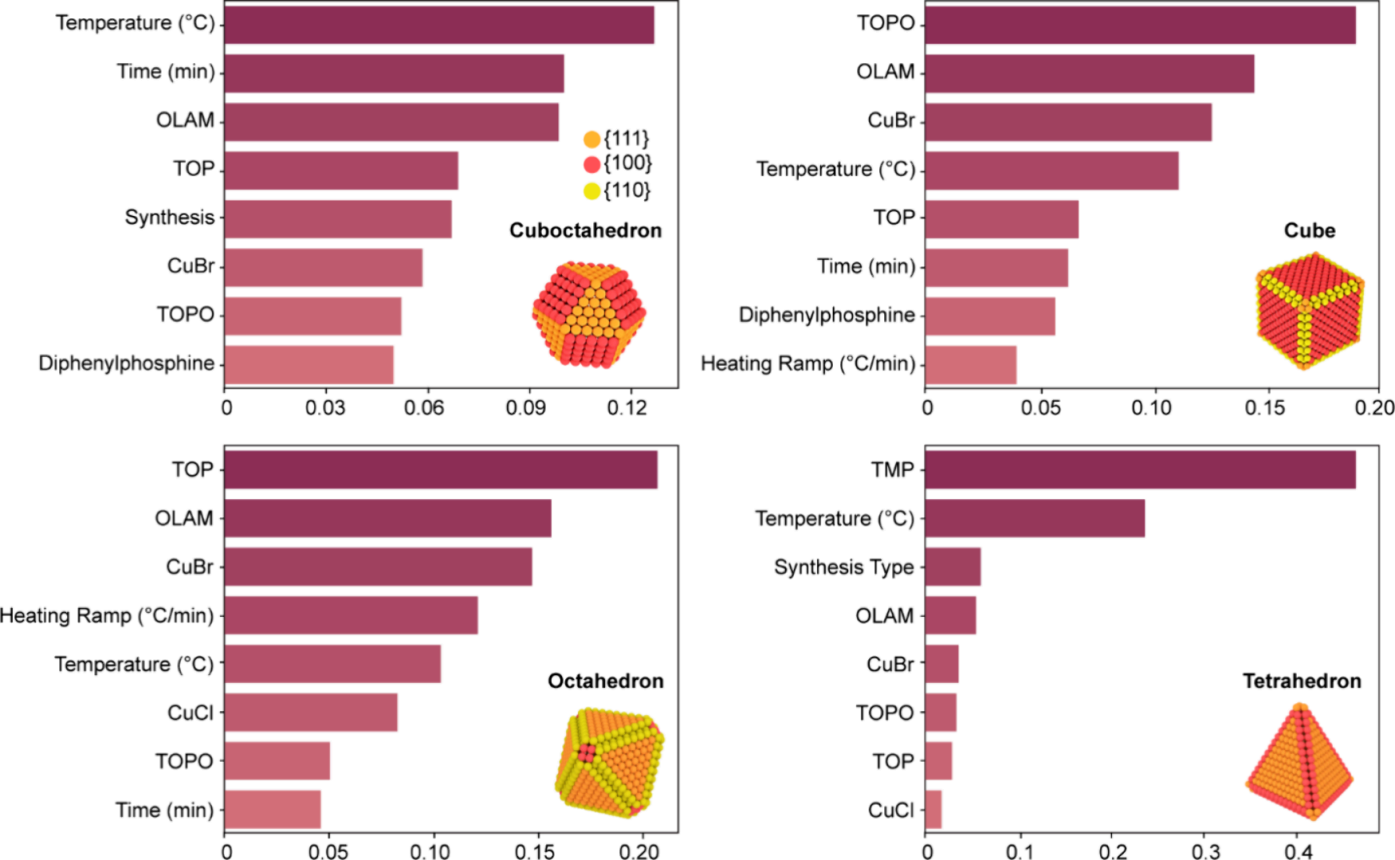
Importance score of different parameters for the synthesis of Cu NCs obtained from the random forest model. Data are reported for spheres (i.e., cuboctahedra), cubes, octahedra, and tetrahedra with corresponding schematic representation of the shapes. Color coding for the shape is orange, red, and yellow for {111}, {100}, and {110} facets, respectively. The abbreviations are CuBr = copper(I) bromide, CuCl = copper(I) chloride, OLAM = oleylamine, TMP = trimethylphosphite, TOP = trioctylphosphine, and TOPO = trioctylphosphine oxide.

The final NC shape typically depends on the precursor reduction kinetics and the presence of a capping agent stabilizing specific crystallographic facets.^1,34,35^ These parameters are largely influenced by the chemicals used (precursors, additives, and solvent) and by the reaction conditions (temperature, heating ramp, and synthesis type). A deep chemical understanding of the most important parameters to target a specific shape usually requires a large number of experiments and the use of in situ techniques.^36−38^ An alternative approach to this traditional process can greatly accelerate the material discovery. Therefore, we combined our data with ML to investigate if meaningful chemical insights could be extracted even with a limited number of data points. After evaluating several models for multilabel binary classification of NC shapes (Table 1), we selected the random forest model for two key reasons. First, the random forest model demonstrated superior predictive performance on our initial data set among tested models (Table 1). Second, the random forest model naturally provides interpretable feature importance measures that quantitatively rank the influence of synthesis parameters on the NC shape outcomes. This interpretability is crucial for gaining chemical insights from model predictions. Then, we extracted and evaluated the critical parameters determining the NC shape in the case of Cu cuboctahedra (generally referred to as “spheres” for their spherical appearance in electron microscopy), cubes, octahedra, and tetrahedra (Figure 2 and Figure S1). Interestingly, the main parameter governing the synthesis of Cu spheres is the reaction temperature. In contrast, chemical reagents emerge as the most important reaction parameters to obtain the other shapes, specifically trioctylphosphine oxide (TOPO) for the cubes, trioctylphosphine (TOP) for the octahedra, and trimethylphosphite (TMP) for the tetrahedra.

**Table 1:** NC Shape Classification Results with Different Classification Models^a^

classifier	precision ↑	recall ↑	F1 score ↑
GPClassifier	0.362 ± 0.053	0.171 ± 0.035	0.225 ± 0.037
logistic regression	0.640 ± 0.121	0.532 ± 0.129	0.560 ± 0.125
SVM	0.630 ± 0.101	0.413 ± 0.070	0.478 ± 0.072
MLP	0.664 ± 0.162	0.592 ± 0.154	0.610 ± 0.158
random forest	**0.702** ± 0.152	0.585 ± 0.168	**0.623** ± 0.154
decision tree	0.635 ± 0.080	**0.604** ± 0.104	0.596 ± 0.066
XGBoost	0.669 ± 0.105	0.505 ± 0.070	0.554 ± 0.073
KNN	0.612 ± 0.125	0.442 ± 0.076	0.486 ± 0.085

a The results include mean and standard deviation across fivefold cross-validation splits over the entire data set.

Overall, these results in Figure 2 match the current understanding of shape control of NCs. For a face-centered cubic (fcc) metal like Cu, the energies of the low-index facets follow the order γ{111} < γ{100} < γ{110}. The sphere, bound by {111} and {100} facets, is the thermodynamically favored shape as it minimizes the total surface free energy. Consequently, Cu spheres can easily be obtained as long as the temperature enables copper formation. No strong chemical dependence is observed apart from oleylamine (OLAM), which is explained by its extensive use in NC synthesis as a solvent, reducing agent, and/or capping agent.^39^ On the other hand, Cu cubes, octahedra, and tetrahedra are kinetic products (i.e., local minima in the free energy landscape) due to a higher overall surface energy compared to spheres.^40^ Therefore, these shapes require a specific chemical potential of the reaction mixture,^38^ which is consistent with the obtained strong dependence on the chemical nature of the reagents. In the case of cubes, the higher-energy {100} facet termination requires a higher monomer flux compared to spheres.^38^ This requirement is only met when CuBr, OLAM, and TOPO are used. The molecular complex formed between CuBr, OLAM, and TOPO rapidly disproportionates at 260 °C, leading to a high Cu(0) flux in solution.^38^ Similarly, a greater monomer flux is achieved by using the disproportionation of CuBr-TOP complexes for octahedra and CuBr-TMP complexes for tetrahedra (Figure S2).^38^

### 2.3 Generating Colloidal Syntheses for Targeted Nanocrystal Shapes Based on a Continuous Energy Scale

Next, we investigated the synthesis of targeted Cu NC shapes. Toward this goal, the use of ML models is challenging because of the low-data regime and the strong correlations between the NC shape and the reaction conditions.^30,38^ To address these challenges, we used Gaussian processes (GPs). While other models (e.g., random forest models) might show higher predictive accuracy, GPs offer the crucial advantage of providing well-calibrated uncertainty estimates alongside their predictions.^33,41^ This uncertainty quantification makes them particularly suitable for guiding experimental optimization, especially in low-data scenarios. GPs are therefore frequent components of BO approaches for optimizing expensive and time-consuming processes such as chemical reactions.^22,42^ The BO strategy uses a GP model as a surrogate model trained on initial data and generates suggestions to be evaluated experimentally (Figure 1).

We tested this approach by describing each NC shape with binary classes (e.g., 1 for present in the synthesis outcome and 0 for absent), and we optimized the reaction conditions to obtain the desired shape. While very successful for generating NC syntheses from a reference library within a given space of NC shapes (Figure S3), the discrete classification prevents the discovery of unknown NC shapes. Therefore, we substituted the binary NC classification with a continuous surface energy scale of different single-crystalline NC shapes to extend the predictive capabilities of the BO model. This approach aims at positioning the different NC shapes on a common continuous scale, thus allowing extrapolation between the reference points. Here, the BO model targets a value of surface energy rather than directly a specific shape. We built the energy scale by computing the surface free energy of NCs in six common single-crystalline shapes (sphere/cuboctahedron, octahedron, cube, rhombic dodecahedron, tetrahedron, and truncated octahedron), each having the same volume. We calculated the surface free energy by determining the surface areas of the {111}, {100}, and {110} facets on each NC with a specific shape and multiplying these by the respective surface energies (see Supplementary Methods for additional details). The surface free energy values of each individual {111}, {100}, and {110} facet are available from the literature.^43,44^

**Figure 3:**
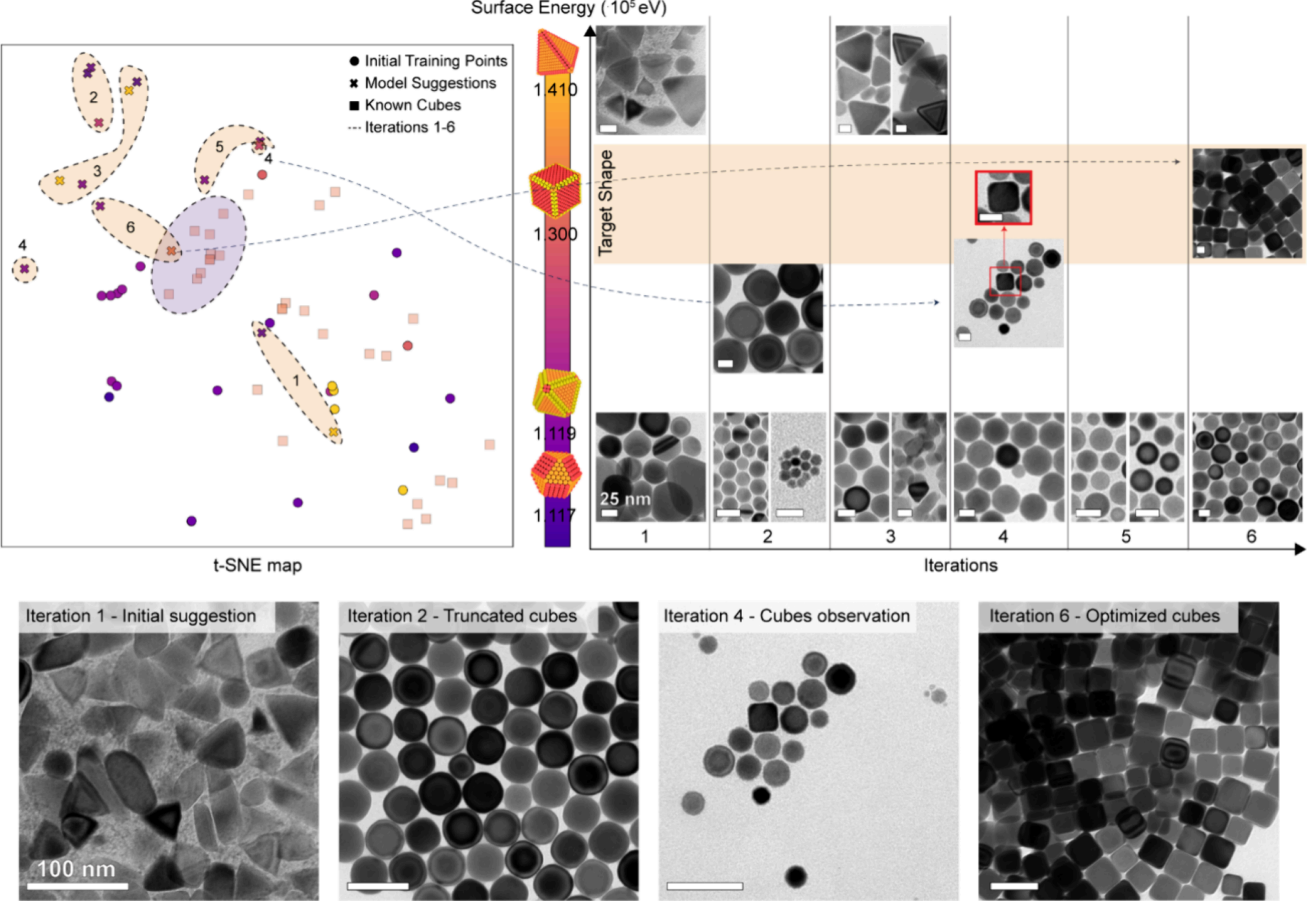
Generated syntheses for targeted NC shapes based on a continuous surface energy scale. In the top left, a 2D map obtained using t-SNE (t-distributed stochastic neighbor embedding) reports different synthesis data points; each point corresponds to a 14-dimensional parameter space including three reaction conditions and 11 reagent values. The data points reported as dots are those used for model training, while the data points marked with “x” are model suggestions. We place the suggestions on the map separated by regions based on the iteration they were proposed in. The points from the data set leading to cubes are reported as square markers; these points were intentionally removed from training to test the model’s nonbiased ability to generate cube recipes. The purple area highlights the high density of data points generating Cu cubes. The color coding of the data points corresponds to the surface energy scale. On the top right, representative TEM images of the obtained NCs are reported for each BO iteration toward the Cu cube NCs given as the target in the energy scale. At the bottom, lower-magnification TEM images are reported for selected iterations. TEM scale bars are 25 nm in the top right panel and 100 nm at the bottom of the figure.

To test our approach, we removed the cubes from our training data and targeted this specific shape through its corresponding value on the surface energy scale via an iterative optimization process (Figure 3). In the first iteration, the model produces recipes for generating mostly tetrahedra (∼60%) and spheres (∼65%). These suggestions are close to the initial training points in the visualized 2D space of reactions. The following iterations place the suggestions into less explored regions, showcasing the explorative nature of the BO process. By the second iteration, we observed the emergence of truncated cubes in high purity (∼95%). Iteration 4 shows the appearance of a small fraction (∼5%) of cubes in the sample and suggests that the model is slowly converging toward the targeted shape. Finally, we obtain cubes in high purity (∼95%) in iteration 6 after only 15 experiments (Figure 3). To put this result into context and highlight the advantages of this approach, the overall size of our design space covers approximately 3 × 10^16^ reactions to explore. By sampling a fraction of this space using BO, we are able to (1) find relevant reactions with targeted outcomes and (2) uncover new reactions that result in other NC shapes. The final optimized suggestion for Cu cubes demonstrates the capabilities of the model to uncover a region containing many options to obtain Cu cubes (purple region in the t-SNE map, Figure 3).

**Figure 4:**
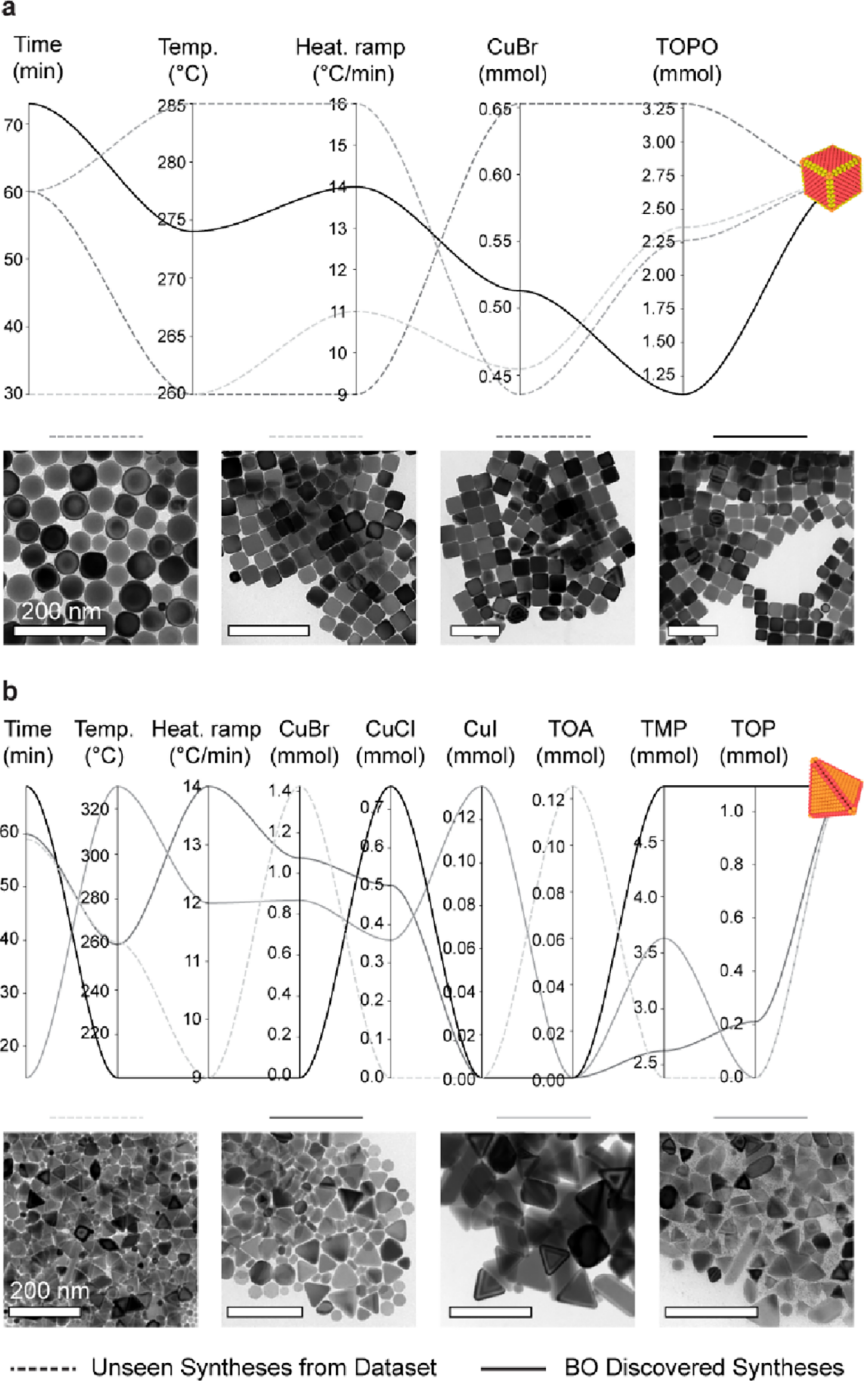
Novel synthetic pathways for cubical and tetrahedral Cu NCs. The parallel coordinate plots report synthetic recipes for (a) Cu cubes and (b) Cu tetrahedra suggested from the BO process (plain lines) compared with a few relevant ones existing in the data set (dashed lines). The existing synthetic pathways for the cubes belong to the purple region of the t-SNE map in Figure 3. The abbreviations are CuBr = copper(I) bromide, CuCl = copper(I) chloride, CuI = copper(I) iodide, TMP = trimethylphosphite, TOA = trioctylamine, TOP = trioctylphosphine, and TOPO = trioctylphosphine oxide.

We note that the proposed synthesis pathways are new and distinct from those included in the original data set (Figure 4), which were hidden from the model. In addition to those for cubes, the explorative nature of the BO process enabled us to uncover new synthesis pathways for other shapes, such as tetrahedral Cu NCs. Notable differences in these syntheses compared to those in the data set include the use of a combination of precursors (CuBr, CuCl, and CuI) and of additives (TOP and TMP), whereas the original syntheses only involved the use of one precursor and one additive. Additionally, the reaction temperatures cover a much wider range spanning from 205 to 330 °C in contrast to 260 °C used originally. These observations underscore various approaches to modulating the chemistry in the solution, leading to the formation of the Cu NCs. Previous studies indicate that CuBr or CuCl and phosphine ligands form molecular complexes that release Cu(0) monomers upon disproportionation.^38,45^ The disproportionation rate of the copper complexes determines the monomer flux and, thus, the shape.

Various correlations can be extracted from Figure 4. Some of these correlations are more trivial. As one example, the reaction kinetics are affected by the precursor concentration (i.e., the disproportionation reaction takes place at a lower temperature when the concentration of copper(I) halides is higher) and the reaction temperature (i.e., the disproportionation reaction is favored at a higher temperature) (Figure 4a). As a second example, the heating ramp has a drastic impact on the monomer formation (i.e., higher heating rates accelerate the conversion of precursor to monomer) (Figure 4a). Beyond, other less trivial correlations emerge. For example, the heterogeneity of the precursor solution impacts the reaction temperature (Figure 4b). In particular, a mixture of CuBr + CuI + CuCl + TMP forms the tetrahedra at higher temperature than CuCl + TMP + TOP and CuBr + TMP. This correlation suggests that different molecular complexes form in solution (Figure S4), and perhaps, this heterogeneity results into a broader disproportionation temperature range, which eventually demands a higher reaction temperature to form a sample with high purity shape. Thus, future efforts should be directed to better understand this aspect and how the different parameters balance out to reach similar chemical potentials that result in the same NC product. Altogether, this information inspires future studies to further our current understanding of the chemistry behind the formation of Cu NCs and, possibly, other non-noble metal NCs.

### 2.4 Discovery of Colloidal Syntheses and Chemistry for New Nanocrystal Shapes

Having demonstrated that the BO model can successfully target the desired NC shape using the continuous surface energy scale, we asked the model to target a shape for which no synthesis exists in the literature. We chose the Cu rhombic dodecahedron (RD). This shape is enclosed by Cu{110} surfaces, thus complementing well the already reported octahedral and cubical Cu NCs, which are bound respectively by {111} and {100} facets. The synthesis of the RD shape has been demonstrated for other fcc metals such as Au or Pd,^46,47^ which indicates the possibility to synthesize this shape for Cu NCs.

**Figure 5:**
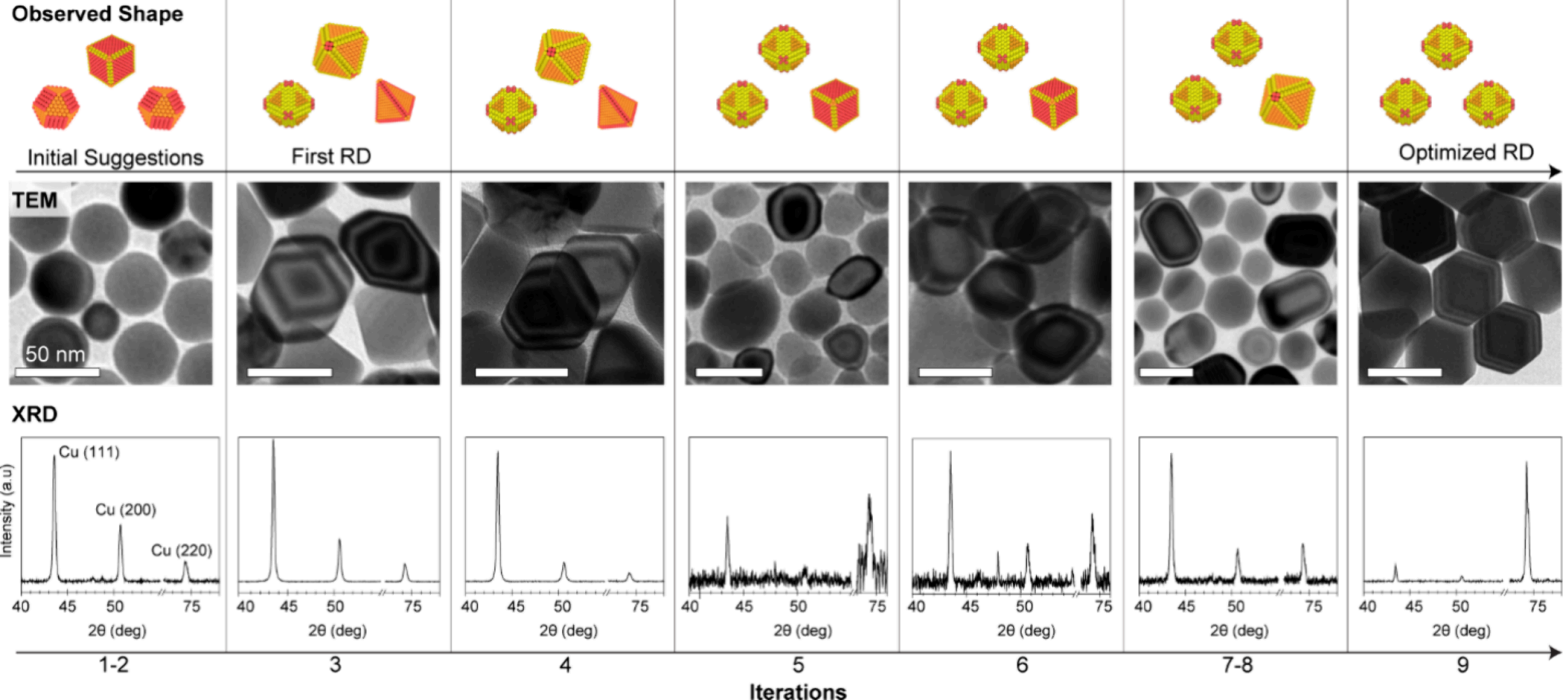
BO-driven synthesis discovery for Cu NCs with a rhombic dodecahedral shape. Representative TEM images and corresponding XRD patterns of the Cu NCs obtained through different BO iterations. The surface energy value corresponding to the RD shape is targeted up to four interactions. After that, the maximization of the Cu(220) reflection in the XRD is added as the second optimization objective. The optimized RD sample is obtained after nine iterations. Scale bars in TEM images are 50 nm.

We proceeded by targeting the value on the surface energy scale corresponding to the RD NCs. While spheres in high purity (∼95%) and truncated cubes in high purity (∼95%) were obtained during the first two iterations, RD NCs appear as a minor fraction of the sample (∼5%) after only three iterations (Figure 5). We note that RD NCs were never observed as impurities in any previous synthesis in our laboratory and in the literature (to the best of our knowledge), which highlights the explorative power of the BO model. Interesting chemical considerations emerge from these results. This first synthesis where RD NCs are observed includes the use of different copper halides and ligands (i.e., CuCl + CuBr + TOP + TMP) (Note S1 and Figures S5–S7). This suggestion is interesting because these ligands are used individually to synthesize octahedra (i.e., CuBr + TOP) and tetrahedra (i.e., CuBr + TMP). In agreement with the insight from Figure 4, the increased solution heterogeneity emerges as a knob to tune the monomer flux. We speculate further that the monomer flux achieved by combining two ligands lies between the individual monomer flux generated by each ligand. A similar reaction outcome is observed when CuI is used in combination with TOP.

At this point, a new challenge has emerged. Indeed, the obtained samples contained a mix of different NC shapes that correspond to different scores on the energy scale. The energy scale optimization alone becomes insufficient because the scale score cannot differentiate between a mixture of two different shapes with high and low values and a pure sample of a shape with an intermediate value. Thus, we added a second optimization objective. This optimization objective aimed at maximizing the intensity of the (220) reflection in XRD, which is indicative of the RD NCs. Having introduced this objective, we obtained one sample of Cu RD NCs as the major fraction (85%) after a total of nine iterations (Figure 5).

**Figure 6:**
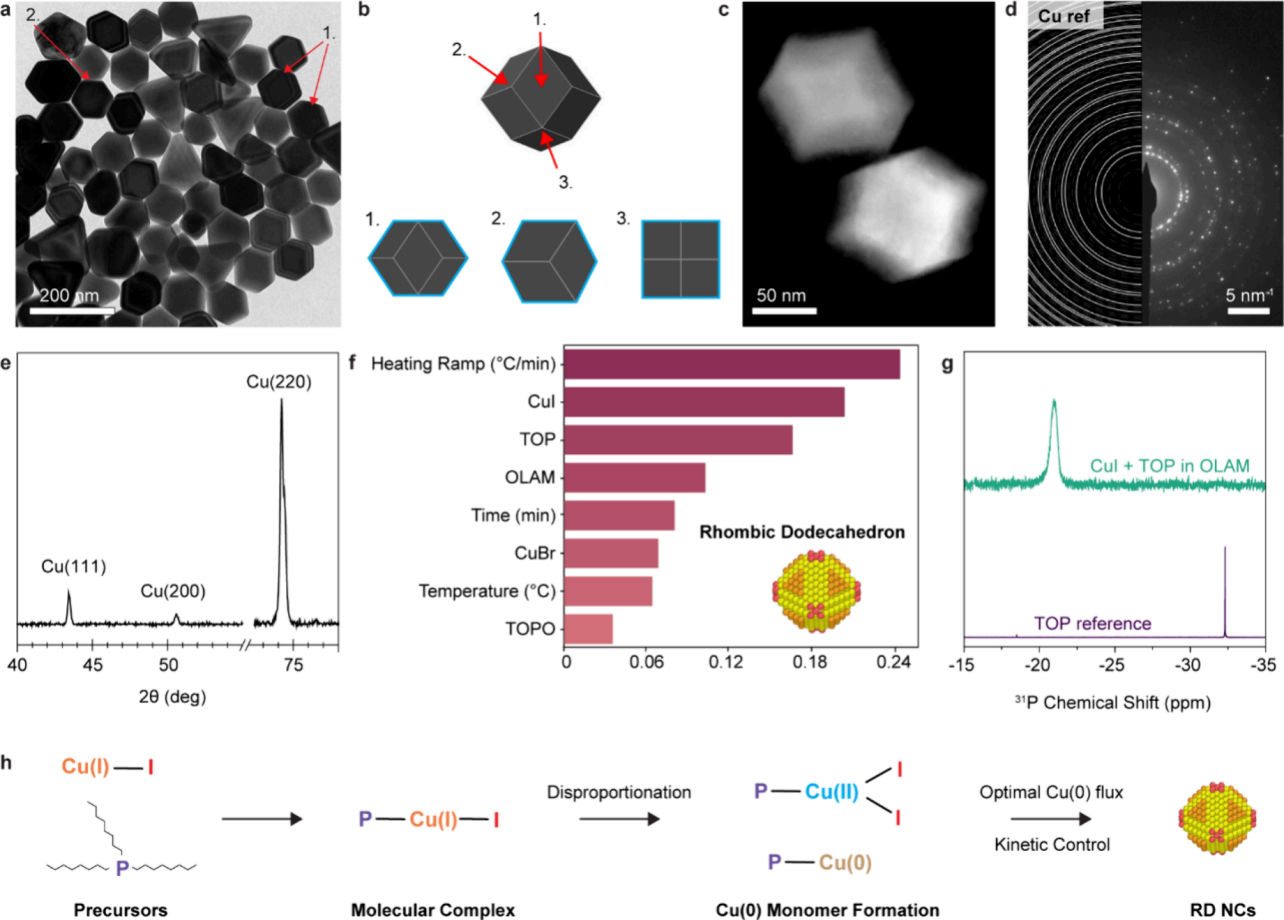
Characterization and formation mechanism of Cu NCs with rhombic dodecahedral shape. (a) Low-magnification TEM image of the optimized Cu RD NCs obtained after nine iterations. The RDs represent ∼85% of the sample. (b) 2D projections of a RD shape from different rotational symmetry axes. (c) HAADF-STEM image of Cu RD NCs highlighting their shape. (d) SAED pattern of the Cu RD NCs with Cu reference. (e) XRD pattern of the Cu RD NCs. (f) Importance score of different parameters for the synthesis of Cu RD NCs obtained from the random forest model similarly to Figure 2. The abbreviations are CuBr = copper(I) bromide, CuI = copper(I) iodide, OLAM = oleylamine, TOP = trioctylphosphine, and TOPO = trioctylphosphine oxide. (g) ^31^P{^1^H} NMR spectrum of an OLAM-CuI-TOP solution (in green) and the TOP reference spectrum (in purple). (h) Proposed reaction mechanism showing how the combination of CuI and TOP led to the formation of Cu RD NCs. The geometry of the molecular complexes is purely hypothetical.

Having obtained this new shape of Cu NCs, we proceeded with a more in-depth structural analysis and chemical analysis. The RD morphology is confirmed by the different 2D projections observed in the TEM and STEM images (Figure 6a–c, Figures S8 and S9). These projections represent ∼85% of the NCs observed in TEM images, whereas the remaining ∼15% are triangular NC byproducts (Figure 6a). The edge length of the synthesized Cu RD NCs is 48.4 ± 4.6 nm (Figure S10). The metallic nature of the NCs is evidenced by selected area electron diffraction (SAED) (Figure 6d) and XRD (Figure 6e). In addition, the XRD pattern shows an intense peak from the Cu(220) reflection and a weak contribution from Cu(111) and Cu(200), which is consistent with the expression of Cu{110} surfaces in the RD NCs (Figure 6e and Figure S11).

The random forest model indicates that the synthesis parameters with the highest importance score are the heating ramp and the use of CuI and TOP (Figure 6f). This chemical information is new because, so far, CuI has not been employed for the controlled and tunable synthesis of Cu NCs in similar reaction environment.^38,45^

^31^P{^1^H} NMR spectroscopy points to a chemical interaction between CuI and TOP (Figure 6g). The phosphorus signal shifts downfield and broadens compared to the free ligand when CuI is mixed with TOP, which is consistent with the formation of a molecular complex, where electron density is transferred from the phosphorus to the Cu atom.

During the synthesis, the solution color shifts from pale yellow to red around 250 °C and later to brown around 265 °C. These color changes are similar to those observed during the disproportionation reaction in the previous examples.^38,45^ Thus, this observation indicates that the CuI-TOP complex might undergo a similar mechanism. We also found a strong dependence on the heating ramp. Specifically, an increased heating rate from ∼4 to ∼25 °C/min generates polyhedral Cu NCs (Figure S12). Interestingly, previous results show no variation in the NC shape when CuCl or CuBr is used.^38^ Here, the substitution of CuI with CuBr switches the NC shape from RDs to cubes (Figure S13). CuBr and CuI behave very differently in the presence of TOPO, used in previous synthesis as well. Specifically, spherical NCs and multiply twinned decahedral/2D stacking fault-lined NCs are obtained with CuBr and CuI, respectively (Figure S14).

Based on these results, we postulate that the combination of CuI and TOP generates the optimal monomer flux to target the metastable RD shape in a kinetic growth regime (Figure 6h). This monomer flux is lower than those achieved with CuBr and CuCl. The slow kinetic crystal growth required for the expression of Cu{110} facets within the single-crystalline shapes is in agreement with the literature for the synthesis of Au RD NCs.^47^ We cannot fully exclude the role of iodine as a selective binding agent of Cu{110}. However, the high sensitivity of the synthesis to the heating ramp suggests that the impact of this factor on the final shape is secondary, if present at all. Actually, we conclude that the critical seeds for the RD shape form in this early step of the synthesis. Then, the growth occurs from here via monomer addition to these seeds, similarly to that proposed for Au RDs.^48^ These observations suggest a new kinetic regime achievable for the synthesis of Cu NCs and define the new conditions and direction to explore toward further synthesis optimization of missing shapes from the library of Cu NCs and beyond, which is in line with further understanding the importance of Cu precursor reactivity in synthesis.^38,45^

## 3 Conclusions

In conclusion, this work proposes a holistic data-driven ML approach that operates in a data-limited scenario to link reaction conditions to nanocrystal shape and the chemistry behind it in colloidal synthesis. We demonstrated how simple predictive models (i.e., random forest) along with iterative methods (i.e., Bayesian optimization) successfully address the low-data regimes associated with colloidal synthesis and, more generally, to fundamental materials chemistry studies. We revealed that the introduction of a continuous scale (i.e., surface energy scale) is crucial for the discovery of synthetic pathways toward materials yet to be reported (i.e., Cu NCs with rhombic dodecahedral shape). We unveil new chemical insights that are promising in driving further the tunability of metal precursor reactivity. Overall, we expect the introduction of this scale to facilitate the translation of the lesson learned from the specific system to others (e.g., NCs with a different crystalline structure) and to other NC properties beyond the shapes.

## 4 Experimental Section

### 4.1 Methods

#### 4.1.1 Data Set Creation

The data collection process is based on the manual extraction of synthetic parameters and reaction outcomes from the literature and our own experiments followed by their integration into an electronic lab notebook (ELN). We have selected an extensive number of synthetic parameters based on their expected impact on the NC shape by influencing the formation kinetics. Additional details are reported in the Supporting Information.

#### 4.1.2 NC Synthetic Parameter Featurization into Machine-Learning Readable Format

The unique feature vectors constitute a high-dimensional space defined by reagent quantities and reaction conditions (temperature, time, heating ramp, etc.). We used an extension of the one-hot-encoding approach to create a unified representation across the data set where the reactions contain multiple reagent combinations. Additional details are reported in the Supporting Information.

#### 4.1.3 Low-Data Machine-Learning Models for Extracting Chemical Insights from NC Synthesis

We tested various low-data-friendly ML models that offer adequate predictive capabilities (i.e., Gaussian processes, logistic regression, support vector machines (SVMs), multilayer perceptrons (MLPs), tree methods, random forest, decision trees and XGBoost, or K-nearest neighbors classifier (KNN)) on our low-data regime data set extracted from the Cu NC syntheses. We used 115 initial data points and 14 occurring shapes to evaluate the performance of the different low-data-friendly ML models. The input space covers 29 reaction parameters including four reaction conditions (time, temperature, heating ramp, and synthesis type) and 25 varying reagents. We observe the random forest model to be leading in both precision and F1 score (Table 1). More importantly, this evaluation validates the practicality of the employed reaction representations and their correlation to the target shapes. Additional details are reported in the Supporting Information.

#### 4.1.4 Bayesian Optimization for Generating Colloidal Syntheses for Targeted Nanocrystal Shapes Using a Multiobjective Optimization

We used BO with a Gaussian process surrogate model over a subset of the initial synthetic parameters. We initially defined our objective function as a multiobjective optimization problem where we maximize the probability of the target shape while minimizing the sum of probabilities of all other shapes. However, due to the limitation of this approach for generating synthesis suggestions for new NC shapes, we introduced a continuous surface energy scale to classify the different NC shapes on a comparable axis, which allowed us to target arbitrary surface energies and their associated NC shapes. Additional details are reported in the Supporting Information.

#### 4.1.5 Continuous Surface Energy Scale Classification of Cu NCs

We constructed the scale assuming a constant NC volume (corresponding to the volume of a 40 nm-edge cube). We calculated the surface free energy by determining the surface areas of the {111}, {100}, and {110} facets for each NC shape and multiplying these by the respective surface energies. The developed continuous scale addresses the limitations of the multiobjective approach by positioning different shapes onto the same scale and allowing us to interpolate between different shapes. Additional details are reported in the Supporting Information.

#### 4.1.6 Bayesian Optimization for Generating Colloidal Syntheses for Targeted Nanocrystal Shapes Using the Continuous Energy Scale

With the energy mapping between the different shapes and their associated surface energies, the objective function of the BO approach is the minimization of the absolute difference between the target surface energy and the surface energy of the resulting shape.

### 4.2 Synthesis of Cu NCs

#### 4.2.1 General Synthetic Procedure

In a glovebox under a N_2_ atmosphere, the copper precursor(s), the additive(s), and the predegassed OLAM solvent were added to a three-neck flask with a stirring magnet. The flask was sealed in the glovebox and quickly connected to a Schlenk line under a N_2_ atmosphere. The reaction mixture was then degassed for 5 min at 60 °C before being heated to the desired reaction temperature (100–330 °C) with the desired heating ramp (2–25 °C/min) during the desired reaction time (2–240 min). At the end of the reaction, the reaction mixture was cooled to approximately 80 °C before being transferred back to the glovebox. The reaction solution was washed and centrifuged two times at 13,000 rpm during 10 min, the first time with 15 mL of hexane and the second time with 5 mL of hexane and 5 mL ethanol. At the end, the final product was collected in 1 mL of toluene for further analysis.

#### 4.2.2 Synthesis of Cu Cubes (Figure 3, Iteration 6)

The general synthetic procedure was followed starting with 73.6 mg of CuBr (0.513 mmol), 430 mg of tri-*n*-octylphosphine oxide (1.112 mmol), and 15.0 mL oleylamine. The reaction temperature was 274 °C, the heating ramp was 14 °C/min, and the reaction time was 73 min.

#### 4.2.3 Synthesis of Cu Rhombic Dodecahedra (Figure 5, Iteration 9)

The general synthetic procedure was followed starting with 83.0 mg of CuI (0.436 mmol), 242 μL of trioctylphosphine (0.543 mmol), and 15.0 mL oleylamine. The reaction temperature was 285 °C, the heating ramp was 4 °C/min, and the reaction time was 60 min.

#### 4.2.4 Other Syntheses

Details about all of the other syntheses can be found in the Supporting Information.

### 4.3 Characterization

#### 4.3.1 Transmission Electron Microscopy (TEM) and Selected Area Electron Diffraction (SAED)

TEM and SAED images were acquired at 120 kV on an FEI Tecnai-Spirit equipped with a Gatan Orius SC200D camera. As-synthesized NCs were drop-cast on a Cu TEM grid (Ted Pella, Inc.) before imaging. Size statistics were determined using ImageJ and counting at least 100 particles per sample. The NC shape was determined manually by counting 100 to 200 particles from different regions of the grid. A specific shape was assigned to the sample based on the major fraction. The SAED patterns were analyzed with the software CrysTBox.^49^ The CIF files used for analysis were downloaded from the Materials Project.^50^

#### 4.3.2 High-Angle Annular Dark Field Scanning Transmission Electron Microscopy (HAADF-STEM)

HAADF-STEM images were acquired on a ThermoFisher Scientific Tecnai Osiris at an accelerating voltage of 200 kV.

#### 4.3.3 X-ray Diffraction (XRD)

XRD measurements were conducted on a Bruker D8 Advance diffractometer with Cu Kα radiation equipped with a Bruker LynxEye 1D energy dispersive detector. The diffractometer operated at 40 kV and 40 mA with a Cu Kα source with a wavelength of λ = 1.54 Å. Samples were prepared by drop-casting nanoparticles on silicon substrates that were cleaned by sonication in hexane. The NC toluene suspensions were drop casted on the substrate, and the toluene was left to evaporate. Serial drops were drop casted to accumulate an adequate amount of NCs for analysis. The XRD spectra shown are voluntarily cropped between 2θ angles of 55 and 72° due to the presence of a very large silicon reflection at 69.5°.

#### 4.3.4 Nuclear Magnetic Resonance Spectroscopy (NMR)

Solution NMR measurements were recorded on a Bruker Avance III HD 400 MHz 9.4 T spectrometer equipped with a BBFO liquid probe. One-dimensional ^31^P spectra were acquired using a standard pulse sequence from the Bruker library. The solvent used for every experiment was toluene-d8.

## Data Availability

All data are available in the main text or the Supporting Information. Experimental data are openly available in Zenodo at 10.5281/zenodo.14823228. The code is available at https://github.com/schwallergroup/boludo.

## Supporting Information Available

Supplementary methods; additional experimental considerations; supplementary notes; additional TEM, SAED, XRD, and size statistics of different Cu NCs; ^31^P{^1^H} experiments of the initial reaction solution; additional characterization of rhombic dodecahedral Cu NCs; and comparison with Cu cubes and Cu octahedra (PDF)
