# Supplementary material for: A Holistic Data-Driven Approach to Synthesis Predictions of Colloidal Nanocrystal Shapes
Source: J Am Chem Soc. 2025 Feb 7;147(7):6116–25. doi: 10.1021/jacs.4c17283 (PMC11848920; doi:10.1021/jacs.4c17283)
Supplement: Supplementary file 1 — ja4c17283_si_001.pdf [file ja4c17283_si_001.pdf]

Supplementary Information

A holistic data-driven approach to synthesis predictions of colloidal nanocrystal shapes

Ludovic Zaza[1+], Bojana Rankovic[2+], Philippe Schwaller[2]*, Raffaella Buonsanti[1]*

[1] Laboratory of Nanochemistry for Energy (LNCE), Department of Chemical Sciences and Engineering, École Polytechnique Fédérale de Lausanne, CH-1950 Sion, Switzerland.

[2] Laboratory of Artificial Chemical Intelligence (LIAC), Department of Chemical Sciences and Engineering, École Polytechnique Fédérale de Lausanne, CH-1015 Lausanne, Switzerland.

[+] These authors contributed equally to this work

* philippe.schwaller@epfl.ch, raffaella.buonsanti@epfl.ch

Table of contents

Methods 3

Additional Experimental Considerations 13

Figure S1 – Importance score of all the different parameters for the synthesis of Cu NCs given in a consistent order. 24

Figure S2 – $^{31}P\{^{1}H\}$ spectra of a solution containing CuBr, OLAM with TOP ligands and with TMP ligands individually. 25

Figure S3 – TEM images of the Cu NCs obtained following the BO model recommendations using a multi-objective optimization for the synthesis of tetrahedra and wires. 26

Figure S4 – Possible impacts of using different copper(I) halide salt precursors and additives on the molecular complexes formed during Cu NC synthesis. 27

Note S1 – Chemical considerations from the BO model suggestions for RD NCs synthesis. 28

Figure S5 – $^{31}P\{^{1}H\}$ spectra of a solution containing CuBr, OLAM with both TOP and TMP ligands. 29

Figure S6 – TEM image and XRD pattern of the NCs obtained in the 3rd iteration in which the first RD Cu NCs were observed. 30

Figure S7 – XRD pattern of the NCs obtained in the 6th iteration when using CuI as a copper precursor. 31

Figure S8 – TEM images of the optimized Cu RD NCs with schematic representation of the NC orientation. 32

Figure S9 –TEM images of Cu RD NCs with different tilt angles. 33

Figure S10 – Size statistics of the optimized Cu RD NCs. 34

Figure S11 – Comparison of the optimized Cu RD NCs XRD pattern with Cu cubes and octahedra XRD patterns. 35

Figure S12 – TEM image of the Cu NCs obtained after heating CuI with TOP in OLAM with a heating ramp of 25°C/min during 60 minutes. 36

Figure S13 – TEM image of the Cu NCs obtained after heating CuBr with TOP in OLAM in similar conditions to the RD NC synthesis. 37

Figure S14 – TEM images of the Cu NCs obtained after heating CuBr and CuI with TOPO in OLAM in similar conditions to the RD NC synthesis. 38

References 39

Methods

Data-set creation

We considered Cu NC syntheses in organic media from various literature reports.[1–12] Despite the possibility to synthesize Cu NCs in aqueous[13–16] or organic[1–12] media, we focused on the synthesis in an organic solvent under an inert atmosphere due to the tendency of non-noble metals to oxidize, which makes their synthesis in water difficult.[17]

The ELN data captures the composition, morphology and size of the NCs as the reaction outcome, alongside the chemical nature of the reagents, their stoichiometry, the reaction conditions including temperature, time, the heating ramp and the synthesis type (heat-up, hot-injection, slow injection) as the main synthetic variables (when available). We expect these parameters to greatly impact the NC shape due to their impact on the reaction kinetics.[1]

NC synthetic parameters featurization into machine-learning readable format

One-hot encoding is a common data processing technique used to handle categorical data in machine learning.[18] Essentially, it converts categorical variables into a binary format levelling each category into a new column, which uses only 1s and 0s to indicate the presence or absence of a feature.

In the context of our study, we deal with various chemical reagents used in reactions, which are categorical by nature. In traditional data formats, three possible reagents: A, B and C can be listed in a single column, with each reaction noting what reagent was used. However, machine learning models require numerical input, so we use one-hot encoding to transform the 'reagent' column into three separate columns: one for each reagent. In these new columns, a ‘1’ indicates that the reagent was used in a reaction, while a '0' indicates it was not. We additionally extend this concept by filling in the values for each reagent using their actual quantities stepping further from binary labelling.

In this regard, we identified all unique reagents present in the dataset and constructed a feature vector by explicitly recording the presence and quantity of each reagent. Additionally, we merged the reagent features with the reaction conditions recorded as numerical and categorical values to create the complete ML-ready reaction representation. Using this format, we set the foundation for applying ML techniques to uncover patterns and correlations between the reaction parameters and the resulting NC shapes.

Low-data machine-learning models for extracting chemical insights from NC synthesis

For the evaluation of predictive capacities of ML models on the generated NC dataset, we used models generally well calibrated for the low-data regime. These models include:

- Gaussian processes:[19] A family of probabilistic models that map relationships between the input and the output as a distribution over functions. Their main advantage is the well-defined access to uncertainty estimates and the output predictions, which makes them a common choice as surrogate models in Bayesian optimization.
- Tree methods
    - Decision trees:[20] A non-parametric supervised learning method used for classification and regression. The model predicts the value of a target variable by learning simple decision rules inferred from the data features. Decision trees are easy to understand and interpret and are adaptable to solving any problem that requires a decision-like structure, including feature importance evaluation.
    - Random forest:[21] A versatile ensemble learning method that constructs multiple decision trees during training and outputs the mode of the classes (classification) or mean prediction (regression) of the individual trees. Random forests are often surprisingly successful at handling low-data offering

robustness by averaging multiple deep decision trees, each trained on different parts of the same training set.

  - XGBoost:[22] An implementation of gradient boosted decision trees designed for speed and performance. XGBoost is renowned for its efficiency at scale and its ability to handle sparse data. It applies a strong regularization technique to reduce overfitting, which is why it performs exceptionally well on various predictive modelling tasks. XGBoost also provides several advanced features for model tuning, including handling missing values, tree pruning, and built-in cross-validation.

- SVM:[23] A powerful classifier that works by finding a hyperplane that best divides a dataset into classes. SVM is effective in high-dimensional spaces and with cases where the number of dimensions exceeds the number of samples. It is also versatile, as different kernel functions can be specified for the decision function. Common kernels include linear, polynomial, radial basis function (RBF), and sigmoid.
- KNN:[24] A simple, instance-based learning algorithm where the new cases are predicted based on a similarity measure (e.g. distance functions). KNN has been used in statistical estimation and pattern recognition as a non-parametric technique. It is particularly effective if the decision boundary is very irregular.
- MLP:[25] A class of feedforward artificial neural network (ANN). An MLP consists of at least three layers of nodes: an input layer, a hidden layer, and an output layer. Except for the input nodes, each node is a neuron that uses a nonlinear activation function. MLP utilizes a technique called backpropagation for training the network. MLPs are suitable for complex decision boundaries and large datasets, making them versatile for a wide range of classification and regression problems.

Alongside input featurization and the choice of the appropriate model, we additionally featurized the reaction outputs (i.e. the NC shape). To do so, we converted the descriptive reactions outcomes from the ELNs into a binary multi-label vector covering fourteen different possible shapes (i.e. assigning 0s or 1s to the synthesis output based on multiple resulting shapes it produces). This approach allows to consider scenarios in which a reaction results in a mixture of NC shapes by labeling all occurring shapes. For example, in a hypothetical reaction resulting in a mixture of NCs, such as cubes and octahedra, out of three available shapes (cube, octahedron, sphere), the outcome would be represented with the binary vector [1, 1, 0], describing the exact combination of shapes in the outcome mixture.

Bayesian optimization for generating colloidal syntheses for targeted nanocrystal shapes using a multi-objective optimization

We used Rational Quadratic and Matern kernel from Gpytorch library[26] and the implementation of a custom GP model on top of SingleTaskGP class from Botorch.[27] We explored Upper Confidence Bound (beta 1.96) and Expected Improvement acquisition functions.

We narrowed down the number of possible reagents to 11 with their quantities ranging from 0 to 5 mmoles, while fixing the synthesis type to a heat-up synthesis, therefore reducing the number of initial conditions to temperature (120-290°C), time (2-150 min) and heating ramp (3-22°C/min). This initial design space encompasses approximately $\sim 3 \cdot 10^{16}$ combinations which we estimate by discretizing temperature (per 10°C), time (per 5 min), heating ramp (per 1°C/min) and reagent quantities (per 0.1 mmol). During the optimization process, we dynamically expanded this search space by extending the parameter boundaries, ultimately exploring temperatures of 100-330°C, heating ramps of 3-25°C/min, reaction times of 2-240 min, and reagent quantities up to 10 mmoles.

We used an unconstrained BO approach by sampling 300’000 Sobol-generated points within the defined design space bounds, from which we selected the top-performing candidates based on the acquisition function values. Subsequently, we refined our search by performing multi-start optimization using either batched expected improvement acquisition function or a Kriging strategy.[28] We designed batches of four experiments to match our daily experimental maximum.

We initially defined an objective function as a multi-objective optimization problem. As previously stated, the reaction outcomes were featurized with binary multi-label vectors where each label can be seen as a separate optimization objective with the goal of maximizing the target label. In this sense, we converted the multiple objectives to a single value maximizing the probability of the target shape while minimizing the sum of probabilities of all other shapes. As an optimization objective, we used a variant of a scaled multi-objective optimization approach where we maximize the class of the targeting shape while minimizing the sum of all other. If a reaction results in multiple simultaneously occurring shapes, each one gets assigned class 1. All the other shapes known and present in the data would be assigned class 0 for that specific reaction outcome. When we are targeting a specific shape from our database, we sum the values associated with that shape in the binary target vector of our data points and subtract the sum of all the other possible target shapes known in the data. As an example, let us consider a database containing four known shapes: cube, sphere, tetrahedron and octahedron.

Our target vector would have the following form:

Shapes	Cube	Sphere	Tetrahedron	Octahedron
Possible values:	(0,1)	(0,1)	(0,1)	(0,1)

For a reaction that results in a cube, this vector would have the form [1,0,0,0], while a reaction with both cube and sphere in the output would take the following format: [1,1,0,0]. If we are

optimizing for cubes in this dataset, we would convert these target vectors into scalar values by subtracting the sum of all other values in the target vectors from the targeted shape column. In the example of cubes this would be: 1-(0+0+0) for the first reaction and 1-(1+0+0) for the second example reaction. This allows us to convert the binary target vectors into scalar values for each reaction and look for a reaction that maximizes their value using Bayesian optimization. With this approach, we aimed to uncover reaction conditions to target specific Cu shapes in the database. We specifically targeted Cu wires and tetrahedra, for which only few data points were included in the initial database. In addition, we did not have any prior experience in their synthesis, which limits biases and ensures that our investigation is purely data-driven. The first iteration of the BO loop already generated experimentally-validated suggestions (**Figure S3**). We took this as a proof of concept that the generated synthetic representations compressed into vector form provide enough input for the model to generate meaningful suggestions. In those experiments, we used Rational Quadratic kernel with Upper Confidence Bound with beta set to 1.96. Having successfully generated the targeted morphologies in the first try we have not explored more parameters.

The main issue with the multi-objective optimization is the limited use case for generating synthesis suggestions for shapes not already known. We overcame this obstacle by introducing a continuous surface energy scale.

Continuous surface energy scale classification of Cu NCs

We used arbitrarily the volume of a 40nm-edge cube (i.e. 64’000 nm^3) as a basis for the calculation of the surface energy of different NC shapes. We used this volume to back-calculate the total surface area of six different single-crystalline NC shapes (sphere, octahedron, cube, rhombic dodecahedron, tetrahedron and truncated octahedron). We did not consider other shapes containing twin defects such as decahedra, wires, plates, etc. due to size-dependent

strain effects.[29,30] The total surface area of each NC was then multiplied by the corresponding energy of the crystalline facets covering the NC surface (i.e. {111} for octahedron, {100} for cube, {110} for rhombic dodecahedron, etc.) to generate a continuous scale classifying the different NC morphologies. As a simplification, we assume the NCs to be perfect single-crystals (i.e. with no truncation at the edges/corners) which allows to express most of the shapes as only being constituted of a single crystallographic facet. For example, with this simplification, cubes are only bound by {100} surfaces, octahedra and tetrahedra by {111} surfaces and rhombic decahedra by {110} surfaces. The two major exceptions are cuboctahedra and truncated octahedra which are both constituted of {111} and {100} surfaces. The ratio of {111} to {100} surfaces is 3.46 for the truncated octahedron and 0.58 for the cuboctahedron (see calculations below).

We used a constant NC volume (V) to keep the bulk free energy constant (i.e. the $\Delta G_V \cdot V$ term in the equation below). In this case, the total free energy of the NC, which is the sum of the surface free energy (ΔG_S) and the bulk free energy scales with the surface free energy.[31]

$$\Delta G = \Delta G_s + V \cdot \Delta G_v$$

We calculated the edge length of each NC morphology with the following mathematic relationships.

1. $e_{cube} = \sqrt[3]{V} = 40.0\,nm$
2. $e_{octahedron} = \sqrt[3]{\frac{3V}{\sqrt{2}}} = 51.4\,nm$
3. $e_{tetrahedron} = \sqrt[3]{6V\sqrt{2}} = 81.6\,nm$
4. $e_{cuboctahedron} = \sqrt[3]{\frac{3V}{5\sqrt{2}}} = 30.1\,nm$
5. $e_{rhombic\ dodecahedron} = \sqrt[3]{\frac{9V}{16\sqrt{3}}} = 27.5\,nm$
6. $e_{truncated\ octahedron} = \sqrt[3]{\frac{V}{8\sqrt{2}}} = 17.8\,nm$

We calculated the surface area of each NC morphology with the following relationships based on the edge lengths.

1. $A_{cube(100)} = 6{e_{cube}}^2 = 9600\ nm^2$
2. $A_{octahedron(111)} = 2\sqrt{3}{e_{octahedron}}^2 = 9151\ nm^2$
3. $A_{tetrahedron(111)} = \sqrt{3}{e_{tetrahedron}}^2 = 11'529\ nm^2$
4. $A_{cuboctahedron(111)} = 2\sqrt{3}{e_{cuboctahedron}}^2 = 3129\ nm^2$
   $A_{cuboctahedron(100)} = 6{e_{cuboctahedron}}^2 = 5420\ nm^2$
5. $A_{rhombic\ dodecahedron(110)} = 8\sqrt{2}{e_{rhombic\ dodecahedron}}^2 = 8553\ nm^2$
6. $A_{truncated\ octahedron(111)} = 12\sqrt{3}{e_{truncated\ octahedron}}^2 = 6599\ nm^2$
   $A_{truncated\ octahedron(100)} = 6{e_{truncated\ octahedron}}^2 = 1905\ nm^2$

We calculated the surface free energy of each NC morphology with the following relationships based on the surface area of the NC and on the surface energy of the low index facets {111}, {100} and {110}. The surface free energy considered were : γ{111} = 1.95 J/m^2, γ{100} = 2.17 J/m^2 and γ{110} = 2.24 J/m^2.[32,33]

1. $\Delta G_{s,cube} = A_{cube(100)}\gamma_{(100)} = 1.300 \cdot 10^5\ eV$
2. $\Delta G_{s,octahedron} = A_{octahedron(111)}\gamma_{(111)} = 1.119 \cdot 10^5\ eV$
3. $\Delta G_{s,tetrahedron} = A_{tetrahedron(111)}\gamma_{(111)} = 1.410 \cdot 10^5\ eV$
4. $\Delta G_{s,cuboctahedron} = A_{cuboctahedron(111)}\gamma_{(111)} + A_{cuboctahedron(100)}\gamma_{(100)} = 1.117 \cdot 10^5\ eV$
5. $\Delta G_{s,rhombic\ dodecahedron} = A_{rhombic\ dodecahedron(110)}\gamma_{(110)} = 1.196 \cdot 10^5\ eV$
6. $\Delta G_{s,truncated\ octahedron} = A_{truncated\ octahedron(111)}\gamma_{(111)} + A_{truncated\ octahedron(100)}\ \gamma_{(100)} = 1.065 \cdot 10^5\ eV$

These values allow to classify the NCs on a common scale based on their surface free energy.

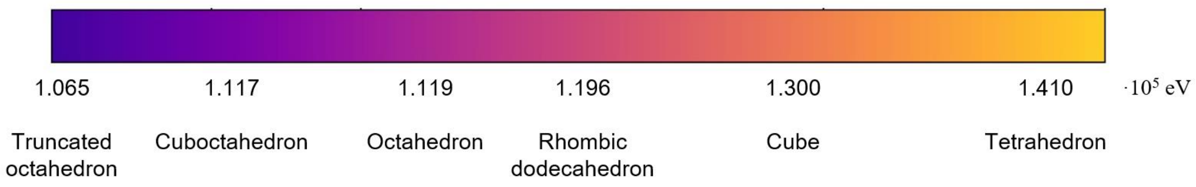

Bayesian optimization for generating colloidal syntheses for targeted nanocrystal shapes using the continuous energy scale

We used Rational Quadratic and Matern kernel from Gpytorch library[26] and the implementation of a custom GP model on top of SingleTaskGP class from Botorch.[27] We explored Upper Confidence Bound (beta 1.96) and Expected Improvement acquisition functions. We started with Rational Quadratic Kernel and Expected improvement acquisition function.

We used the Cu cube synthesis as a testing ground for different parameters of the surrogate model. In the third iteration, we switched the acquisition function to Upper confidence bound. We used parallel batch optimization from Botorch suggesting from 4 to 8 reactions per iteration. Depending on the daily budget and available resources we selected 2 to 4 reactions out of these suggestions to test them in the lab. We note that with the sufficient budget this additional constraint would not be necessary as it would be possible to experimentally evaluate all suggestions. In the last iteration, we changed the kernel to Matern and generated 8 suggestions out of which we chose 2, one of which resulted in the target shape.

For the rhombic dodecahedron synthesis, we kept the previous parameters (Matern kernel with Expected improvement acquisition function). After observing RDs in the third BO iteration, we defined the resulting scale value as an average of the observed shape mixture in the sample (tetrahedra, octahedra, RD). However, we saw little progress with this approach. Therefore, we tested the binary labelling method used for Cu wires and tetrahedra since the RD shape was present in the dataset (cf. Bayesian optimization for generating colloidal syntheses for targeted nanocrystal shapes using a multi-objective optimization). However, the objective function value was too noisy and, instead, we generated proportions of different shapes in the mixture by using XRD data. We compared the ratios of the Cu(111), Cu(200) and Cu(220) reflections, and estimated proportions for each of the shapes present in the mixture. Using these

proportions, we calculated a weighted average of the scale values to obtain a more fine-grained result that would better compare different mixture outputs. After reaching more than 10 samples with RDs present in the mixture, we converted the optimization method towards maximizing the (220) reflection in the XRD, and used only the RD samples as an input to the model to generate suggestions.

Additional Experimental Considerations

Chemicals

All chemicals were used as received, with no further purification. Copper(I) bromide (CuBr, 99.999% trace metals basis), copper(I) chloride (CuCl, ReagentPlus®, purified, ≥99%), copper(I) iodide (CuI, 99.999% trace metals basis), copper(II) acetate ($Cu(OAc)_2$, 99.99% trace metals basis), tri-n-octylphosphine oxide (TOPO, $O=P(oct)_3$, 99% ReagentPlus®), triphenylphosphine (PPh_3, ReagentPlus® 99%), trimethylphosphite (TMP, $P(OMe)_3$, ≥ 99%), diphenylphosphine ($HPPh_2$, 98%), oleylamine (OLAM, technical grade, 70%), dodecylamine (98%), trioctylamine (TOA, 98%), nitric acid (70%), toluene-d8 (99 atom %D), toluene (anhydrous, 99.8%) and ethanol (dried, max 0.01% H_2O) were purchased from Sigma-Aldrich. Hexane (anhydrous, 96%) was purchased from TCI. Tri-n-octylphosphine (TOP, $P(oct)_3$, technical grade, 90%), triphenylphosphite ($P(OPh)_3$, 97%) and tris(2,4,6-trimethylphenyl)phosphine ($P(mesityl)_3$, 98%) were purchased from Thermoscientific Alfa Aesar.

General synthesis considerations

All syntheses and manipulations of Cu NCs were done under a dry N_2 atmosphere, using Schlenk-line techniques or a glovebox. Anhydrous organic solvents were used for the manipulation, analysis and storage of Cu NCs. All glassware was oven-dried prior to use. Concentrated nitric acid was used to remove any metallic residues from the reaction flask after each reaction and the flask was then washed thoroughly with ultra-pure water prior to oven drying. A J-KEM Scientific Model 310 temperature controller was used with a heating mantle for reaction temperature control.

Synthesis of Cu NCs from model suggestions using a multi-objective optimization

Synthesis of Cu tetrahedra (Figure S3A)

The general synthetic procedure was followed starting with 85.9 mg CuBr (0.599 mmol), 118 μL trimethylphosphite (1.001 mmol), 23 μL trioctylamine (0.053 mmol) and 6.3 mL oleylamine. The reaction temperature was 261°C, the heating ramp was 9°C/min and the reaction time was 59 minutes.

Synthesis of Cu wires (Figure S3B)

The general synthetic procedure was followed starting with 59.4 mg CuCl (0.600 mmol), 2.5 mg triphenylphosphine (0.001 mmol), 384.5 mg tris(2,4,6-trimethylphenyl)phosphine (0.990 mmol), 21 mg tri-n-octylphosphine oxide (0.054 mmol), 3.5 μL trioctylphosphine (0.008 mmol) and 7.4 mL oleylamine. The reaction temperature was 272°C, the heating ramp was 16°C/min and the reaction time was 84 minutes.

Synthesis of Cu cube NCs from model suggestions using the energy scale

Iteration 1, Synthesis 1 (Initial suggestion shown in Figure 3)

The general synthetic procedure was followed starting with 26.5 mg CuCl (0.268 mmol), 82.2 mg CuBr (0.573 mmol), 50 μL trioctylphosphine (0.112 mmol), 165 μL trimethylphosphite (1.399 mmol) and 8.0 mL oleylamine. The reaction temperature was 260°C, the heating ramp was 14°C/min and the reaction time was 60 minutes.

Iteration 1, Synthesis 2

The general synthetic procedure was followed starting with 22.2 mg CuBr (0.155 mmol), 1320 mg tri-n-octylphosphine oxide (3.41 mmol), 95.2 μL dodecylamine (0.414 mmol) and 10.3 ml oleylamine. The reaction temperature was 278°C, the heating ramp was 14°C/min and the reaction time was 64 minutes.

Iteration 2, Synthesis 1 (Truncated cube shown in Figure 3)

The general synthetic procedure was followed starting with 138.0 mg CuBr (0.962 mmol), 87.0 mg CuCl (0.879 mmol), 200 μL trioctylphosphine (0.448 mmol), 773 μL trimethylphosphite (6.55 mmol) and 15 ml oleylamine. The reaction temperature was 244°C, the heating ramp was 12°C/min and the reaction time was 57 minutes.

Iteration 2, Synthesis 2

The general synthetic procedure was followed starting with 149.0 mg CuBr (1.04 mmol), 82.2 mg CuCl (0.828 mmol), 62 mg CuI (0.326 mmol), 177.5 mg copper(II) acetate (0.977 mmol), 2344 mg tri-n-octylphosphine oxide (6.06 mmol), 1328 μL trioctylphosphine (2.98 mmol), 610 μL trimethylphosphite (5.17 mmol) and 15 ml oleylamine. The reaction temperature was 300°C, the heating ramp was 8°C/min and the reaction time was 55 minutes.

Iteration 2, Synthesis 3

The general synthetic procedure was followed starting with 146.0 mg CuBr (1.02 mmol), 85.0 mg CuCl (0.859 mmol), 196.0 mg copper(II) acetate (1.08 mmol), 3060 mg tri-n-octylphosphine oxide (7.91 mmol), 1043 μL trioctylphosphine (2.34 mmol), 725 μL trimethylphosphite (6.15 mmol) and 15 ml oleylamine. The reaction temperature was 212°C, the heating ramp was 11°C/min and the reaction time was 78 minutes.

Iteration 2, Synthesis 4

The general synthetic procedure was followed starting with 136.0 mg CuBr (0.948 mmol), 59.0 mg CuCl (0.596 mmol), 69.0 mg CuI (0.362 mmol), 89.0 mg copper(II) acetate (0.490 mmol), 2040 mg tri-n-octylphosphine oxide (5.28 mmol), 915 μL trioctylphosphine (2.05 mmol), 725 μL trimethylphosphite (6.15 mmol) and 15 ml oleylamine. The reaction temperature was 210°C, the heating ramp was 10°C/min and the reaction time was 74 minutes.

Iteration 3, Synthesis 1

The general synthetic procedure was followed starting with 124.5 mg CuBr (0.868 mmol), 35.6 mg CuCl (0.360 mmol), 25.8 mg CuI (0.135 mmol), 428 μL trimethylphosphite (3.63 mmol) and 15 ml oleylamine. The reaction temperature was 330°C, the heating ramp was 12°C/min and the reaction time was 14 minutes.

Iteration 3, Synthesis 2

The general synthetic procedure was followed starting with 68.8 mg CuBr (0.480 mmol), 51.0 mg CuCl (0.515 mmol), 178 μL trioctylphosphine (0.399 mmol), 541 μL trimethylphosphite (4.59 mmol) and 15 ml oleylamine. The reaction temperature was 311°C, the heating ramp was 15°C/min and the reaction time was 61 minutes.

Iteration 3, Synthesis 3

The general synthetic procedure was followed starting with 75.3 mg CuCl (0.761 mmol), 488 μL trioctylphosphine (1.09 mmol), 588 μL trimethylphosphite (4.99 mmol) and 15 ml oleylamine. The reaction temperature was 201°C, the heating ramp was 9°C/min and the reaction time was 69 minutes.

Iteration 3, Synthesis 4

The general synthetic procedure was followed starting with 47.5 mg CuBr (0.331 mmol), 60.9 mg CuCl (0.596 mmol), 48.5 mg CuI (0.255 mmol), 870 μL trioctylphosphine (1.95 mmol), 268 μL trimethylphosphite (2.27 mmol) and 15 ml oleylamine. The reaction temperature was 216°C, the heating ramp was 12°C/min and the reaction time was 93 minutes.

Iteration 4, Synthesis 1 (Cubes observation sample shown in Figure 3)

The general synthetic procedure was followed starting with 100.8 mg CuBr (0.703 mmol), 1124 mg tri-n-octylphosphine oxide (2.91 mmol), 120 μL trimethylphosphite (1.02 mmol) and 15 ml oleylamine. The reaction temperature was 290°C, the heating ramp was 10°C/min and the reaction time was 141 minutes.

Iteration 4, Synthesis 2

The general synthetic procedure was followed starting with 86.5 mg CuBr (0.603 mmol), 57.3 mg CuCl (0.579 mmol), 138 mg tri-n-octylphosphine oxide (0.357 mmol), 285 μL trioctylphosphine (0.639 mmol), 202 μL trimethylphosphite (1.71 mmol) and 15 ml oleylamine. The reaction temperature was 279°C, the heating ramp was 28°C/min and the reaction time was 167 minutes.

Iteration 5, Synthesis 1

The general synthetic procedure was followed starting with 85.8 mg CuBr (0.598 mmol), 1551 mg tri-n-octylphosphine oxide (4.01 mmol), 124 μL trimethylphosphite (1.05 mmol) and 15 ml oleylamine. The reaction temperature was 254°C, the heating ramp was 7°C/min and the reaction time was 72 minutes.

Iteration 5, Synthesis 2

The general synthetic procedure was followed starting with 85.8 mg CuBr (0.598 mmol), 1551 mg tri-n-octylphosphine oxide (4.01 mmol), 124 μL trimethylphosphite (1.05 mmol) and 15 ml oleylamine. The reaction temperature was 254°C, the heating ramp was 7°C/min and the reaction time was 150 minutes.

Iteration 6, Synthesis 1 (Optimized cubes from Figure 3)

The general synthetic procedure was followed starting with 73.6 mg CuBr (0.513 mmol), 430 mg tri-n-octylphosphine oxide (1.11 mmol) and 15 ml oleylamine. The reaction temperature was 274°C, the heating ramp was 14°C/min and the reaction time was 73 minutes.

Iteration 6, Synthesis 2

The general synthetic procedure was followed starting with 73.6 mg CuBr (0.513 mmol), 29.0 mg CuCl (0.293 mmol), 62.0 mg copper(II) acetate (0.341 mmol), 850 mg tri-n-octylphosphine

oxide (2.20 mmol), 422 μL trioctylphosphine (0.946 mmol), 118 μL trimethylphosphite (1.00 mmol) and 15 ml oleylamine. The reaction temperature was 310°C, the heating ramp was 13°C/min and the reaction time was 50 minutes.

Synthesis of Cu RD NCs from model suggestions using the energy scale

Iteration 1, Synthesis 1

The general synthetic procedure was followed starting with 122.0 mg CuBr (0.850 mmol), 45.0 mg CuCl (0.455 mmol), 59.5 mg triphenylphosphine (0.227 mmol), 825 μL trioctylphosphine (1.85 mmol), 2400 mg tri-n-octylphosphine oxide (6.21 mmol), 42 μL trioctylamine (0.096 mmol), 76 μL trimethylphosphite (0.644 mmol) and 10 ml oleylamine. The reaction temperature was 260°C, the heating ramp was 21°C/min and the reaction time was 81 minutes.

Iteration 2, Synthesis 1

The general synthetic procedure was followed starting with 77.9 mg CuBr (0.543 mmol), 333 μL trioctylphosphine (0.747 mmol), 934 mg tri-n-octylphosphine oxide (2.42 mmol) and 15 ml oleylamine. The reaction temperature was 303°C, the heating ramp was 5°C/min and the reaction time was 30 minutes.

Iteration 2, Synthesis 2

The general synthetic procedure was followed starting with 124.9 mg CuCl (1.26 mmol), 1000 μL trioctylphosphine (2.24 mmol), and 15 ml oleylamine. The reaction temperature was 258°C, the heating ramp was 6°C/min and the reaction time was 73 minutes.

Iteration 3, Synthesis 1 (First synthesis of RD NCs)

The general synthetic procedure was followed starting with 137.0 mg CuBr (0.955 mmol), 88.3 mg CuCl (0.892 mmol), 348 μL trioctylphosphine (0.780 mmol), 739 μL trimethylphosphite

(6.27 mmol) and 15 ml oleylamine. The reaction temperature was 257°C, the heating ramp was 11°C/min and the reaction time was 64 minutes.

Iteration 3, Synthesis 2

The general synthetic procedure was followed starting with 131.4 mg CuCl (1.33 mmol), 1000 µL trioctylphosphine (2.24 mmol), and 15 ml oleylamine. The reaction temperature was 258°C, the heating ramp was 6°C/min and the reaction time was 73 minutes.

Iteration 3, Synthesis 3

The general synthetic procedure was followed starting with 86.2 mg CuBr (0.601 mmol), 268 mg tri-n-octylphosphine oxide (0.693 mmol) and 15 ml oleylamine. The reaction temperature was 202°C, the heating ramp was 14°C/min and the reaction time was 158 minutes.

Iteration 4, Synthesis 1

The general synthetic procedure was followed starting with 187.0 mg CuBr (1.30 mmol), 153.0 mg CuCl (1.55 mmol), 550 µL trioctylphosphine (1.23 mmol), 916 µL trimethylphosphite (7.77 mmol) and 15 ml oleylamine. The reaction temperature was 299°C, the heating ramp was 11°C/min and the reaction time was 69 minutes.

Iteration 4, Synthesis 2

The general synthetic procedure was followed starting with 145.3 mg CuBr (1.01 mmol), 86.4 mg CuCl (0.873 mmol), 346 µL trioctylphosphine (0.776 mmol), 346 µL trimethylphosphite (2.93 mmol) and 15 ml oleylamine. The reaction temperature was 277°C, the heating ramp was 12°C/min and the reaction time was 76 minutes.

Iteration 5, Synthesis 1

The general synthetic procedure was followed starting with 137.6 mg CuBr (0.959 mmol), 89.6 mg CuCl (0.905 mmol), 277 μL trioctylphosphine (0.622 mmol), 749 μL trimethylphosphite (6.35 mmol) and 15 ml oleylamine. The reaction temperature was 257°C, the heating ramp was 12°C/min and the reaction time was 64 minutes.

Iteration 5, Synthesis 2

The general synthetic procedure was followed starting with 157.7 mg CuCl (1.59 mmol), 1429 mg tri-n-octylphosphine oxide (3.70 mmol) and 15 ml oleylamine. The reaction temperature was 282°C, the heating ramp was 12°C/min and the reaction time was 25 minutes.

Iteration 5, Synthesis 3

The general synthetic procedure was followed starting with 139.4 mg CuCl (1.41 mmol), 997 μL trioctylphosphine (2.24 mmol) and 15 ml oleylamine. The reaction temperature was 258°C, the heating ramp was 4°C/min and the reaction time was 73 minutes.

Iteration 5, Synthesis 4

The general synthetic procedure was followed starting with 248.2 mg CuI (1.30 mmol), 802 μL trioctylphosphine (1.80 mmol) and 15 ml oleylamine. The reaction temperature was 261°C, the heating ramp was 5°C/min and the reaction time was 65 minutes.

Iteration 6, Synthesis 1

The general synthetic procedure was followed starting with 144.1 mg CuBr (1.00 mmol), 92.0 mg CuCl (0.929 mmol), 374 μL trioctylphosphine (0.839 mmol), 360 μL trimethylphosphite (3.05 mmol) and 15 ml oleylamine. The reaction temperature was 271°C, the heating ramp was 12°C/min and the reaction time was 67 minutes.

Iteration 6, Synthesis 2

The general synthetic procedure was followed starting with 89.2 mg CuI (0.468 mmol), 221 μL trioctylphosphine (0.496 mmol) and 15 ml oleylamine. The reaction temperature was 284°C, the heating ramp was 3°C/min and the reaction time was 50 minutes.

Iteration 6, Synthesis 3

The general synthetic procedure was followed starting with 126.7 mg CuCl (1.28 mmol), 336 mg tri-n-octylphosphine oxide (0.869 mmol), 1270 μL trioctylphosphine (2.85 mmol) and 15 ml oleylamine. The reaction temperature was 267°C, the heating ramp was 4°C/min and the reaction time was 69 minutes.

Iteration 6, Synthesis 4

The general synthetic procedure was followed starting with 132.0 mg CuCl (1.33 mmol), 998 μL trioctylphosphine (2.24 mmol) and 15 ml oleylamine. The reaction temperature was 261°C, the heating ramp was 5°C/min and the reaction time was 73 minutes.

Iteration 7, Synthesis 1

The general synthetic procedure was followed starting with 115.0 mg CuI (0.604 mmol), 304 μL trioctylphosphine (0.682 mmol), 942 mg tri-n-octylphosphine oxide (2.44 mmol) and 15 ml oleylamine. The reaction temperature was 300°C, the heating ramp was 5°C/min and the reaction time was 30 minutes.

Iteration 7, Synthesis 2

The general synthetic procedure was followed starting with 89.0 mg CuI (0.467 mmol), 235 μL trioctylphosphine (0.527 mmol) and 15 ml oleylamine. The reaction temperature was 265°C, the heating ramp was 3°C/min and the reaction time was 45 minutes.

Iteration 7, Synthesis 3

The general synthetic procedure was followed starting with 138.3 mg CuBr (0.964 mmol), 90.0 mg CuCl (0.909 mmol), 400 μL trioctylphosphine (0.897 mmol), 564 μL trimethylphosphite (4.78 mmol) and 15 ml oleylamine. The reaction temperature was 239°C, the heating ramp was 4°C/min and the reaction time was 49 minutes.

Iteration 7, Synthesis 4

The general synthetic procedure was followed starting with 109.2 mg CuBr (0.761 mmol), 172 μL trioctylphosphine (0.385 mmol) and 15 ml oleylamine. The reaction temperature was 259°C, the heating ramp was 26°C/min and the reaction time was 44 minutes.

Iteration 8, Synthesis 1

The general synthetic procedure was followed starting with 89.0 mg CuI (0.467 mmol), 235 μL trioctylphosphine (0.527 mmol) and 15 ml oleylamine. The reaction temperature was 295°C, the heating ramp was 3°C/min and the reaction time was 45 minutes.

Iteration 8, Synthesis 2

The general synthetic procedure was followed starting with 138.3 mg CuBr (0.964 mmol), 90.0 mg CuCl (0.909 mmol), 400 μL trioctylphosphine (0.897 mmol), 564 μL trimethylphosphite (4.78 mmol) and 15 ml oleylamine. The reaction temperature was 215°C, the heating ramp was 7°C/min and the reaction time was 49 minutes.

Iteration 8, Synthesis 3

The general synthetic procedure was followed starting with 70.0 mg CuBr (0.488 mmol), 270 μL trioctylphosphine (0.605 mmol) and 15 ml oleylamine. The reaction temperature was 270°C, the heating ramp was 24°C/min and the reaction time was 44 minutes.

Iteration 8, Synthesis 4

The general synthetic procedure was followed starting with 70.0 mg CuBr (0.488 mmol), 270 μL trioctylphosphine (0.605 mmol) and 15 ml oleylamine. The reaction temperature was 270°C, the heating ramp was 27°C/min and the reaction time was 44 minutes.

Iteration 9, Synthesis 1

The general synthetic procedure was followed starting with 90.6 mg CuI (0.476 mmol), 217 μL trioctylphosphine (0.487 mmol) and 15 ml oleylamine. The reaction temperature was 283°C, the heating ramp was 3°C/min and the reaction time was 51 minutes.

Iteration 9, Synthesis 2

The general synthetic procedure was followed starting with 82.9 mg CuI (0.435 mmol), 228 μL trioctylphosphine (0.511 mmol) and 15 ml oleylamine. The reaction temperature was 285°C, the heating ramp was 3°C/min and the reaction time was 52 minutes.

Iteration 9, Synthesis 3

The general synthetic procedure was followed starting with 74.4 mg CuI (0.391 mmol), 326 μL trioctylphosphine (0.731 mmol), 252 mg tri-n-octylphosphine oxide (0.652 mmol) and 15 ml oleylamine. The reaction temperature was 284°C, the heating ramp was 3°C/min and the reaction time was 48 minutes.

Iteration 9, Synthesis 4 (Optimized RD sample from Figure 5)

The general synthetic procedure was followed starting with 83.0 mg CuI (0.436 mmol), 242 μL trioctylphosphine (0.543 mmol) and 15 ml oleylamine. The reaction temperature was 285°C, the heating ramp was 4°C/min and the reaction time was 60 minutes.

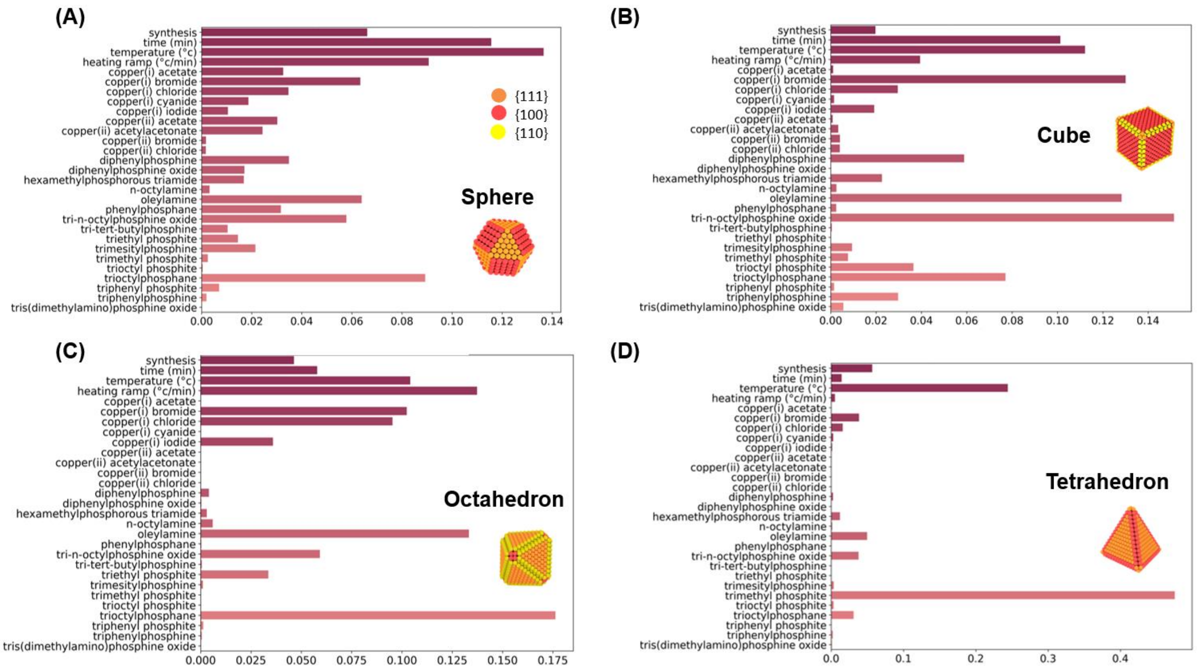

Figure S1. Importance score of all the different parameters for the synthesis of Cu NCs given in a consistent order.

In contrast to the system-centric view in the main paper, here we provide a comprehensive comparison showing how synthesis parameters affect different Cu NC morphologies. By presenting all parameters in a consistent order across shapes, we can observe how the same parameter may significantly influence one morphology while having minimal impact on another. For instance, some parameters that appear prominently for tetrahedra (such as trimethyl phosphite) show different levels of importance for other shapes like cubes, octahedra and spheres. This complete ranking helps understand the complex interplay between synthesis conditions and shape control across different NC morphologies.

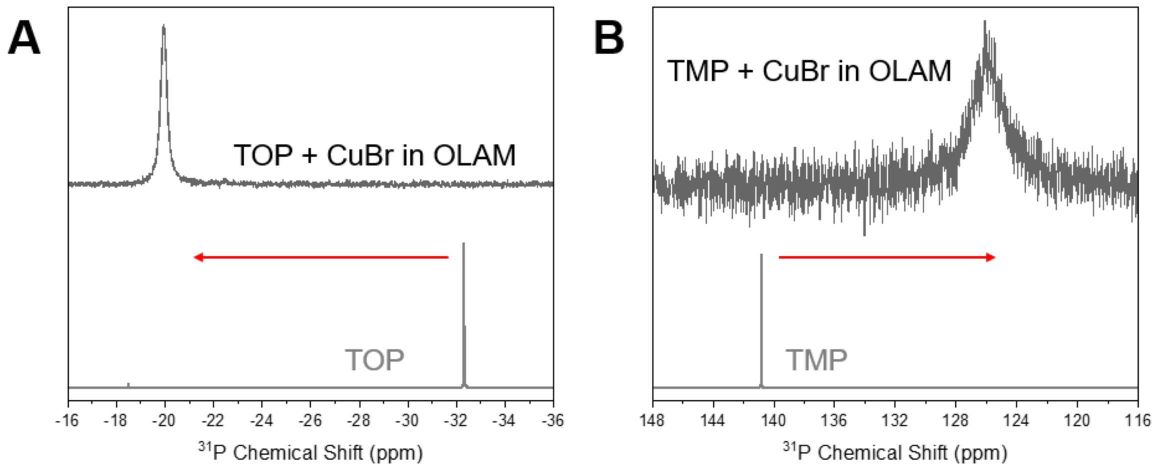

Figure S2. $^{31}P\{^1H\}$ NMR spectrum of an (A) OLAM-CuBr-TOP solution in toluene d8 (black, top) compared to TOP reference (grey, bottom) and (B) OLAM-CuBr-TMP solution in toluene d8 (black, top) compared to TMP reference (grey, bottom).

Shifts in the phosphorus signals are observed in both cases, which indicate the coordination of the phosphines to the metallic centers. A downfield shift of the phosphorus signal is observed with TOP compared to the free ligand (**Figure S2A**). This shift indicates that the electronic density on the phosphorus atom decreases, which is consistent with the formation of a CuBr-TOP complex where TOP coordinates to the Cu(I) center and electron density is transferred by σ-donation. If the ligand is a good π-acceptor, electron density can also be transferred back from the metallic center, causing an upfield shift in the phosphorus resonance. In this case, the net shift will depend on both the ligand σ-donation and π-backbonding abilities. Because TMP has a much better π-backbonding capability than TOP,[36] and because the influence of π-backbonding is normally significantly greater than for σ-donation in ^{31}P NMR,[37] we observe a net upfield shift with TMP (**Figure S2B**).

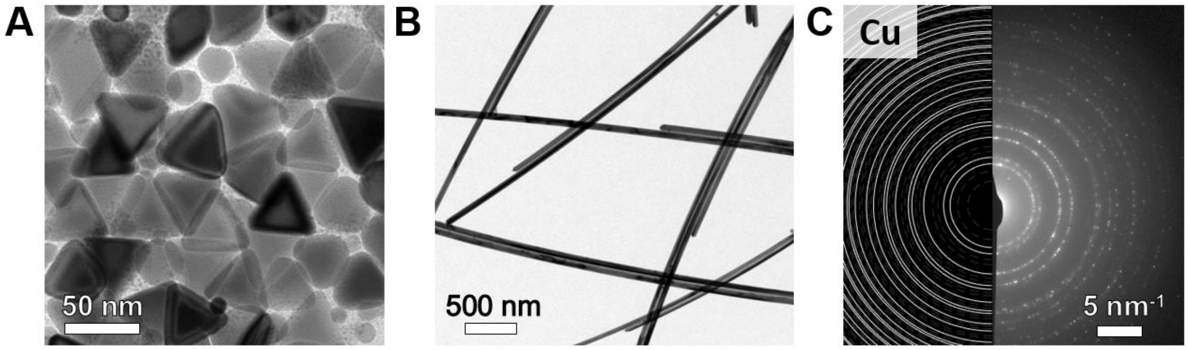

Figure S3. TEM images of the Cu NCs obtained following the BO model recommendations for the synthesis of (A) tetrahedra and (B) wires. (C) Selected area electron diffraction (SAED) of the Cu tetrahedral NCs from (A) with metallic Cu reference for comparison.

Figure S3A and **Figure S3B** show the TEM images of Cu tetrahedra and wires obtained initially by following the BO model recommended experiments using a multi-objective optimization. Selected area electron diffraction (SAED) confirms the metallic nature of the synthesized NCs (**Figure S3C**). The model correctly identifies the important reagents and their quantities to synthesize the targeted NCs. This result underlies the model's capacity to uncover critical regions of the high-dimensional design space that contain targeted objectives.

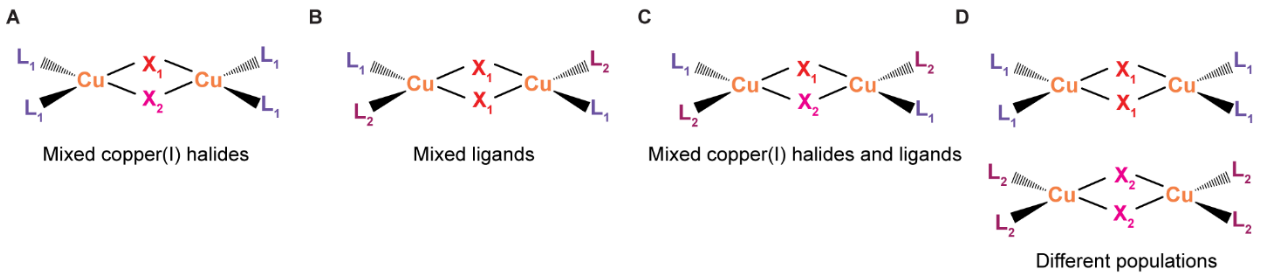

Figure S4. Possible impacts of using different copper(I) halide salt precursors and additives on the molecular complexes formed during Cu NC synthesis. Different possibilities include molecular complexes with: (A) mixed copper(I) halides with the same ligand, (B) mixed ligands with the same copper(I) halide, (C) mixed copper(I) halides with mixed ligands and (D) different independent populations with distinct copper(I) halide and ligands. In the schematics the halides are abbreviated with the letter "X" and the ligands with the letter "L". The geometry of the molecular complexes is purely hypothetical.

Note S1: Chemical considerations from the BO model suggestions for RD NCs synthesis

The first chemical recipes where RD NCs were observed in iterations 3 and 4 includes the combined use of TOP and TMP ligands. This suggestion is interesting because these ligands have been used only individually so far to synthesize octahedra and tetrahedra respectively. The combination of these two ligands indicated by the BO model suggests that the monomer flux can potentially be controlled by combining different additives, which is a new information. Here, RDs (surface energy scale value of ~$1.196 \cdot 10^5$ eV) were synthesized by mixing the ligands used in the synthesis of octahedra (surface energy scale value of ~$1.119 \cdot 10^5$ eV) and of tetrahedra (surface energy scale value of ~$1.410 \cdot 10^5$ eV). $^{31}P\{^1H\}$ NMR spectroscopy indicates that both TOP and TMP coordinate to the copper precursors to form hybrid complexes and/or a population of CuBr-TOP/CuCl-TOP and CuBr-TMP/CuCl-TMP complexes (**Figure S4** and **Figure S5**) which allow to modify the overall Cu flux. However, x-ray diffraction (XRD) analysis of the samples indicate that they are mainly constituted of tetrahedral and octahedral NCs due to the high intensity of the (111) reflection compared to the (220) reflection (**Figure S6**).

In parallel, the model investigates the use of copper(I) iodide as a precursor, which was proposed for one synthesis of tetrahedral NCs (**Figure 4**). Cu RD NCs are also obtained in iteration 5 and beyond by combining CuI and TOP (**Figure 5**). In this case, we observed a substantial increase of the (220) reflection in the XRD, which is associated with the production of more NCs enclosed by {110} facets (**Figure S7**).

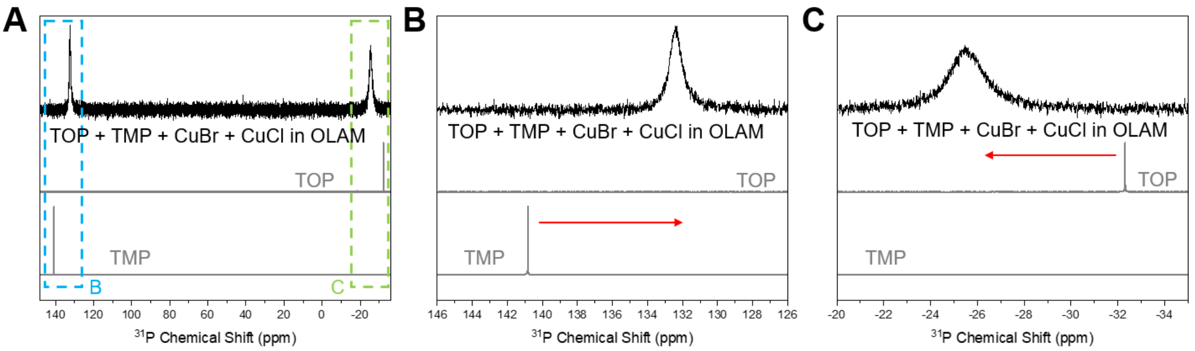

Figure S5. (A) $^{31}P\{^{1}H\}$ NMR spectrum of a solution heated for 5 minutes at 60°C containing CuBr and CuCl mixed with TOP and TMP ligands in OLAM (black, top) compared to TOP reference (grey, middle) and TMP reference (grey, bottom). (B) Zoom in the blue region of Figure S5A. (C) Zoom in the green region of Figure S5A**.**

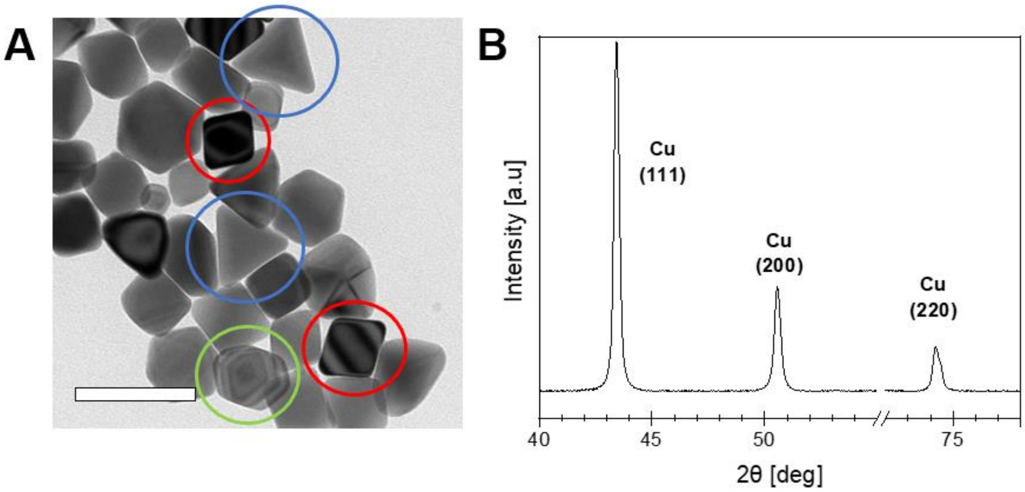

Figure S6. (A) Bright field TEM image of the NCs obtained in the first RD sample. The RD NC (with an elongated hexagonal projection) is highlighted in green and is in minority with respect to octahedra (with a rhombus projection) in red and tetrahedra (with a triangular projection) in blue. Scale bar is 100 nm. (B) XRD pattern of the NCs in (A). XRD agrees with TEM, showing the predominance of octahedra and tetrahedra in the sample which are both enclosed by {111} facets. Instead, the RD which are enclosed by {110} facets (here (220) due to forbidden (110) fcc reflection) facets remains in minority in the sample.

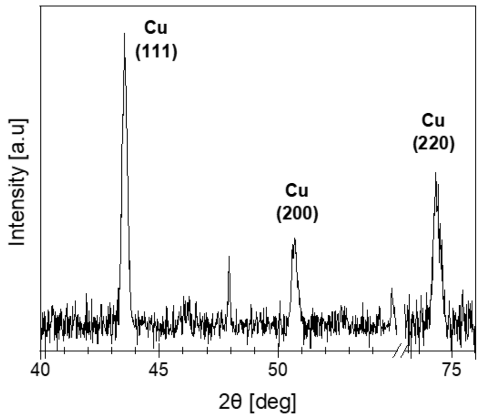

Figure S7. XRD pattern of the NCs shown in Figure 5 (iteration 6) which were synthesized with CuI with an increased contribution of the (220) reflection indicating the formation of more RD NCs.

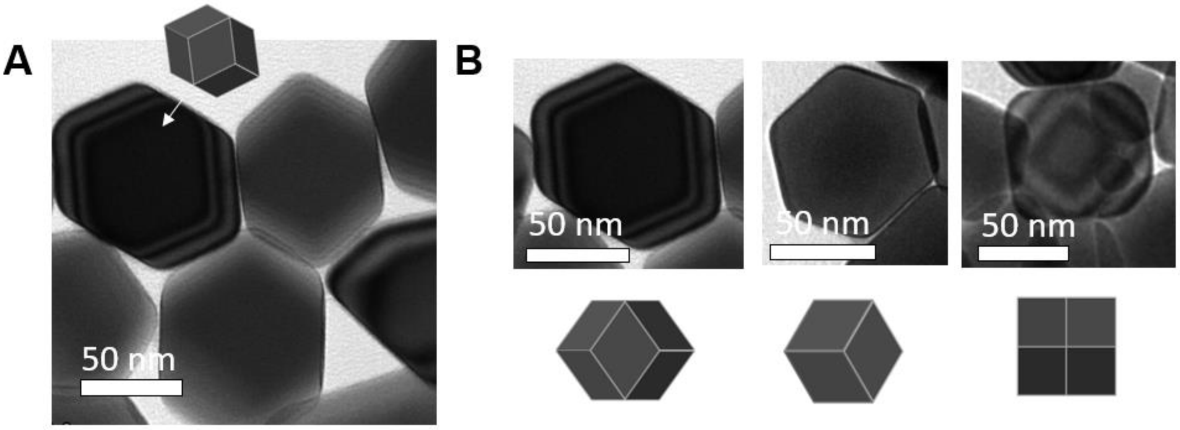

Figure S8. (A) High magnification TEM image of the optimized Cu RD sample with schematic representation of the NC orientation. (B) TEM images of different RD 2D projections.

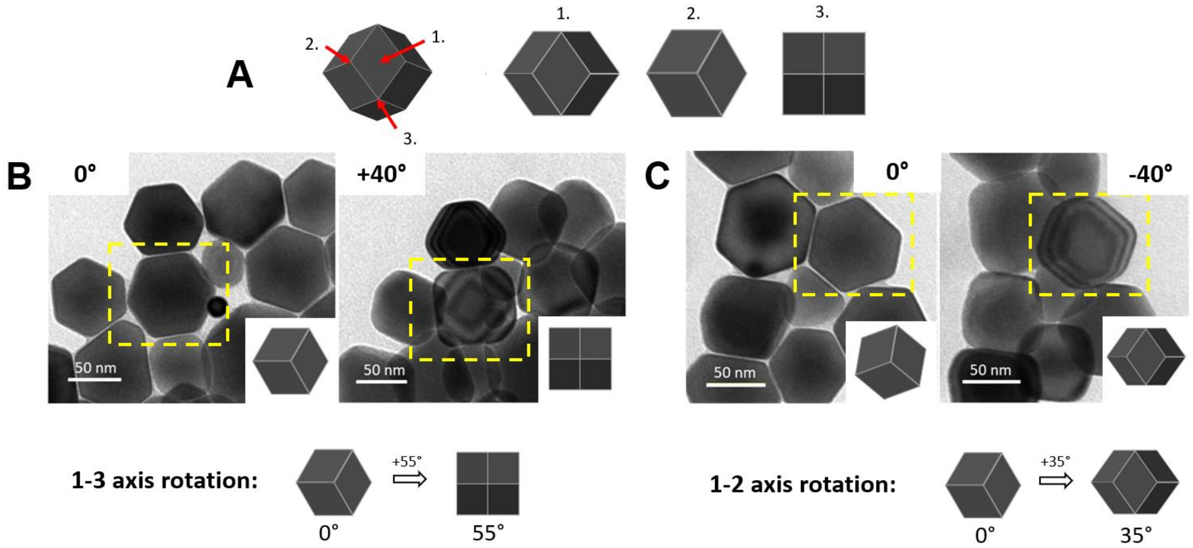

Figure S9. (A) The different 2D projections expected for a RD NC. The NC appears (1) as an elongated hexagon when looked through the twofold axis, which is a line passing through the center of two opposite faces, (2) as a hexagon when looked through the threefold axis, which is a line passing through two opposite vertices where three faces meet, and (3) as a square when looked through the 4-fold axis, which is a line passing through two opposite vertices where four faces meet. (B) One dimensional tilt of a RD NC from 0° to +40° showing the transition from a hexagonal projection (view 2 in A) to a square projection (view 3 in A). This transition between the two projections is expected after a ~55° rotation. (C) One dimensional tilt of a RD NC from 0° to -40° showing the transition from a hexagonal projection (view 2 in A) to an elongated hexagonal projection (view 1 in A). This transition between the two projections is expected after a ~35° rotation.

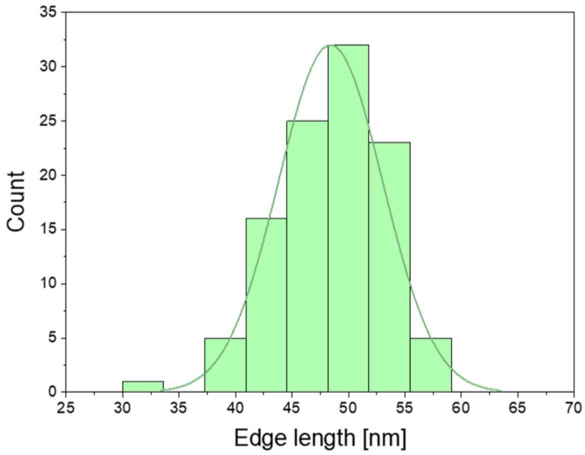

Figure S10. Size distribution of the Cu RD NCs shown in Figure 6a and Figure S8A.

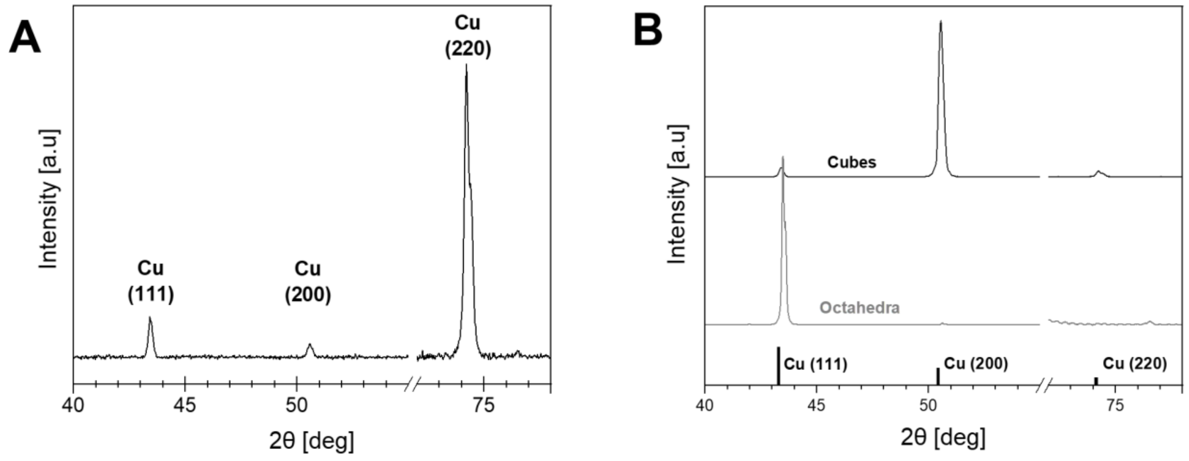

Figure S11. XRD pattern of (A) RD Cu NCs and (B) Cu cubes (in black) and Cu octahedra (in grey) with polycrystalline Cu reference (PDF#00-004-0836).

The {110} termination of the Cu RD NCs is evidenced by XRD. Indeed, the XRD pattern of the Cu RD sample shows an intense peak from the (220) diffraction plane with weak contribution from the (111) and (200) planes (**Figure S11A**). The ratio between the (220) intensity and the (111) and (200) intensities (respectively 7.29 and 21.98) is much higher than what is observed for Cu cubes (0.66 and $3.74 \cdot 10^{-2}$), Cu octahedra ($7.49 \cdot 10^{-3}$ and 0.77) and bulk fcc Cu (0.20 and 0.43) (**Figure S11B**), confirming the presence of {110} facets on the RD NC surface.

Cu cubes and Cu octahedra showcase similar XRD patterns where one peak is much more intense than the others. Cubes have an intense peak for Cu(200) while octahedra have an intense peak for Cu(111) due to the presence of Cu{100} and Cu{111} at their surface respectively.

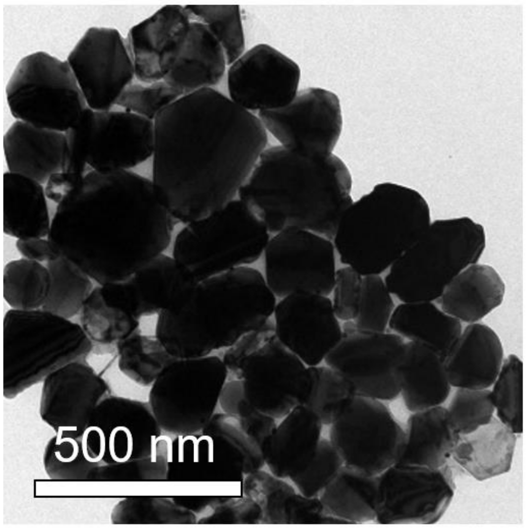

Figure S12. Bright field TEM image of the NCs obtained after heating CuI with TOP in OLAM with a heating ramp of 25°C/min during 60 minutes.

Slow kinetics of NC formation are critical to obtain Cu RD enclosed by {110} facets. The heating ramp must be ~4°C/min to synthesize the RD NCs (**Figure S8**). Indeed, a heating ramp of 25°C/min generates only irregularly shaped NCs in the same conditions (**Figure S12**).

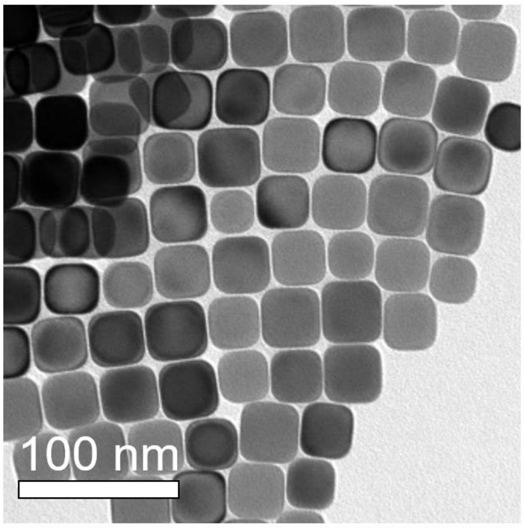

Figure S13. Bright field TEM image of the NCs obtained after heating CuBr with TOP in OLAM in similar conditions to the ones forming Cu RD NCs (Figure S8).

CuI is essential to obtain the optimal slow kinetics for the synthesis of Cu RD NCs (**Figure S8**). Cu cubes are obtained when CuBr is used instead of CuI as a precursor in the same reaction conditions (**Figure S13**).

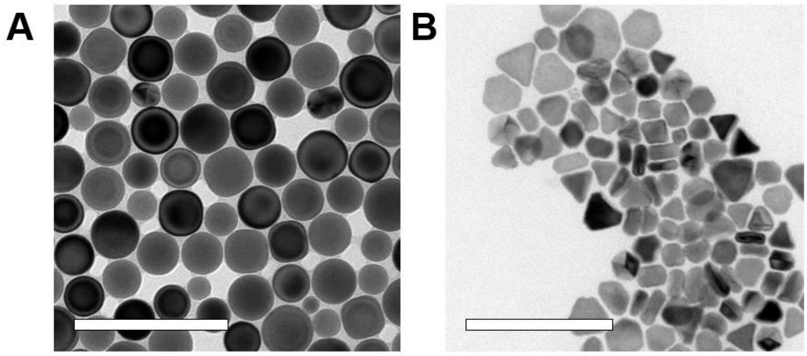

Figure S14. Bright field TEM image of (A) the single-crystalline spherical Cu NCs obtained after heating CuBr with TOPO in OLAM in similar conditions to the ones leading to Cu RD NCs (Figure S8) and (B) the twinned decahedral and 2D stacking fault-lined Cu NCs obtained after heating CuI with TOPO in OLAM in the same conditions. Scale bars are 200 nm.

The crucial role of CuI compared to CuBr is also highlighted when the ligands are changed. For example, the substitution of TOP with TOPO in the same reaction conditions leading to Cu RDs leads to single-crystalline Cu spheres with CuBr (**Figure S14A**) whereas twinned and stacking fault-lined NCs are obtained with CuI (**Figure S14B)**.

Single-crystalline NCs are the typical reaction outcome at higher reduction rates. Instead, the NCs adopt a twinned morphology when the reduction rate is reduced, eventually leading to stacking fault-lined 2D NCs when the rate is very slow.[38–40] Therefore, this result indicates that the monomer flux is lower with CuI than with CuBr.

Reference

(1) Strach, M.; Mantella, V.; Pankhurst, J. R.; Iyengar, P.; Loiudice, A.; Das, S.; Corminboeuf, C.; Van Beek, W.; Buonsanti, R. Insights into Reaction Intermediates to Predict Synthetic Pathways for Shape-Controlled Metal Nanocrystals. *J. Am. Chem. Soc.* **2019**, *141* (41), 16312–16322. https://doi.org/doi.org/10.1021/jacs.9b06267.

(2) Loiudice, A.; Lobaccaro, P.; Kamali, E. A.; Thao, T.; Huang, B. H.; Ager, J. W.; Buonsanti, R. Tailoring Copper Nanocrystals towards C2 Products in Electrochemical CO2 Reduction. *Angew. Chemie - Int. Ed.* **2016**, *55* (19), 5789–5792. https://doi.org/10.1002/anie.201601582.

(3) Iyengar, P.; Kolb, M. J.; Pankhurst, J. R.; Calle-Vallejo, F.; Buonsanti, R. Elucidating the Facet-Dependent Selectivity for CO_2 Electroreduction to Ethanol of Cu–Ag Tandem Catalysts. *ACS Catal.* **2021**, *11* (8), 4456–4463. https://doi.org/10.1021/acscatal.1c00420.

(4) De Trizio, L.; Figuerola, A.; Manna, L.; Genovese, A.; George, C.; Brescia, R.; Saghi, Z.; Simonutti, R.; Van Huis, M.; Falqui, A. Size-Tunable, Hexagonal Plate-like Cu 3P and Janus-like Cu-Cu 3P Nanocrystals. *ACS Nano* **2012**, *6* (1), 32–41. https://doi.org/10.1021/nn203702r.

(5) Iyengar, P.; Huang, J.; De Gregorio, G. L.; Gadiyar, C.; Buonsanti, R. Size Dependent Selectivity of Cu Nano-Octahedra Catalysts for the Electrochemical Reduction of CO_2 to CH_4. *Chem. Commun.* **2019**, *55* (60), 8796–8799. https://doi.org/10.1039/C9CC02522G.

(6) De Gregorio, G. L.; Burdyny, T.; Loiudice, A.; Iyengar, P.; Smith, W. A.; Buonsanti, R. Facet-Dependent Selectivity of Cu Catalysts in Electrochemical CO_2 Reduction at Commercially Viable Current Densities. *ACS Catal.* **2020**, *10* (9), 4854–4862.

https://doi.org/10.1021/acscatal.0c00297.

(7) Lu, S.-C.; Hsiao, M.-C.; Yorulmaz, M.; Wang, L.-Y.; Yang, P.-Y.; Link, S.; Chang, W.-S.; Tuan, H.-Y. Single-Crystalline Copper Nano-Octahedra. *Chem. Mater* **2015**, *27*, 2022. https://doi.org/10.1021/acs.chemmater.5b03519.

(8) Guo, H.; Chen, Y.; Cortie, M. B.; Liu, X.; Xie, Q.; Wang, X.; Peng, D.-L. Shape-Selective Formation of Monodisperse Copper Nanospheres and Nanocubes via Disproportionation Reaction Route and Their Optical Properties. *J. Phys. Chem. C* **2014**, *118* (18), 9801–9808. https://doi.org/10.1021/jp5014187.

(9) Liu, J.; Meyns, M.; Zhang, T.; Arbiol, J.; Cabot, A.; Shavel, A. Triphenyl Phosphite as the Phosphorus Source for the Scalable and Cost-Effective Production of Transition Metal Phosphides. *Chem. Mater.* **2018**, *30* (5), 1799–1807. https://doi.org/10.1021/acs.chemmater.8b00290.

(10) Rachkov, A. G.; Schimpf, A. M. Colloidal Synthesis of Tunable Copper Phosphide Nanocrystals. *Chem. Mater.* **2021**, *33* (4), 1394–1406. https://doi.org/10.1021/acs.chemmater.0c04460.

(11) Suen, N.-T.; Kong, Z.-R.; Hsu, C.-S.; Chen, H.-C.; Tung, C.-W.; Lu, Y.-R.; Dong, C.-L.; Shen, C.-C.; Chung, J.-C.; Chen, H. M. Morphology Manipulation of Copper Nanocrystals and Product Selectivity in the Electrocatalytic Reduction of Carbon Dioxide. *ACS Catal.* **2019**, *9* (6), 5217–5222. https://doi.org/10.1021/acscatal.9b00790.

(12) Ouyang, L. Synthèse de Nanoparticules de Cuivre Par Dismutation de Complexes de Cuivre(I) et Développement de Nouvelles Voies d’accès Par Stratégie Organométallique, 2022.

(13) Jin, M.; He, G.; Zhang, H.; Zeng, J.; Xie, Z.; Xia, Y. Shape-Controlled Synthesis of Copper Nanocrystals in an Aqueous Solution with Glucose as a Reducing Agent and

Hexadecylamine as a Capping Agent. *Angew. Chemie Int. Ed.* **2011**, *50* (45), 10560–10564. https://doi.org/10.1002/anie.201105539.

(14) Salzemann, C.; Urban, J.; Lisiecki, I.; Pileni, M.-P. Characterization and Growth Process of Copper Nanodisks. *Adv. Funct. Mater.* **2005**, *15* (8), 1277–1284. https://doi.org/10.1002/adfm.200400594.

(15) Sun, Y.; Xu, L.; Yin, Z.; Song, X. Synthesis of Copper Submicro/Nanoplates with High Stability and Their Recyclable Superior Catalytic Activity towards 4-Nitrophenol Reduction. *J. Mater. Chem. A* **2013**, *1* (39), 12361. https://doi.org/10.1039/c3ta12526b.

(16) Lee, J.-W.; Han, J.; Lee, D. S.; Bae, S.; Lee, S. H.; Lee, S.-K.; Moon, B. J.; Choi, C.-J.; Wang, G.; Kim, T.-W. 2D Single-Crystalline Copper Nanoplates as a Conductive Filler for Electronic Ink Applications. *Small* **2018**, *14* (8), 1703312. https://doi.org/10.1002/smll.201703312.

(17) Mantella, V.; Castilla-Amorós, L.; Buonsanti, R. Shaping Non-Noble Metal Nanocrystals *via* Colloidal Chemistry. *Chem. Sci.* **2020**, *11* (42), 11394–11403. https://doi.org/10.1039/D0SC03663C.

(18) James, G.; Witten, D.; Hastie, T.; Tibshirani, R. *An Introduction to Statistical Learning*; Springer Texts in Statistics; Springer US: New York, NY, 2021. https://doi.org/10.1007/978-1-0716-1418-1.

(19) Rasmussen, C. E.; Williams, C. K. I. *Gaussian Processes for Machine Learning*; The MIT Press: Cambridge, 2005. https://doi.org/10.7551/mitpress/3206.001.0001.

(20) Breiman, L.; Friedman, J. H.; Olshen, R. A.; Stone, C. J. *Classification And Regression Trees*; Routledge, 2017. https://doi.org/10.1201/9781315139470.

(21) Breiman, L. Random Forests. *Mach. Learn.* **2001**, *45*, 5–32. https://doi.org/10.1023/A:1010933404324.

(22) Chen, T.; Guestrin, C. XGBoost. In *Proceedings of the 22nd ACM SIGKDD International Conference on Knowledge Discovery and Data Mining*; ACM: New York, NY, USA, 2016; pp 785–794. https://doi.org/10.1145/2939672.2939785.

(23) Cortes, C.; Vapnik, V. Support-Vector Networks. *Mach. Learn.* **1995**, *20* (3), 273–297. https://doi.org/10.1007/BF00994018.

(24) Cover, T.; Hart, P. Nearest Neighbor Pattern Classification. *IEEE Trans. Inf. Theory* **1967**, *13* (1), 21–27. https://doi.org/10.1109/TIT.1967.1053964.

(25) Haykin, S. *Neural Networks: A Comprehensive Foundation*; Prentice Hall PTR, 1994.

(26) Gardner, J. R.; Pleiss, G.; Bindel, D.; Weinberger, K. Q.; Wilson, A. G. GPyTorch: Blackbox Matrix-Matrix Gaussian Process Inference with GPU Acceleration. In *NIPS'18: Proceedings of the 32nd International Conference on Neural Information Processing Systems*; 2018; pp 7587–7597. https://doi.org/10.48550/arXiv.1809.11165.

(27) Balandat, M.; Karrer, B.; Jiang, D. R.; Daulton, S.; Letham, B.; Wilson, A. G.; Bakshy, E. BOTORCH: A Framework for Efficient Monte-Carlo Bayesian Optimization. In *NIPS'20: Proceedings of the 34th International Conference on Neural Information Processing Systems*; 2020; pp 21524–21538. https://doi.org/10.48550/arXiv.1910.06403.

(28) Ginsbourger, D.; Le Riche, R.; Carraro, L. Kriging Is Well-Suited to Parallelize Optimization; 2010; pp 131–162. https://doi.org/10.1007/978-3-642-10701-6_6.

(29) Xia, Y.; Xiong, Y.; Lim, B.; Skrabalak, S. E. Shape-Controlled Synthesis of Metal Nanocrystals: Simple Chemistry Meets Complex Physics? *Angew. Chemie Int. Ed.* **2009**, *48* (1), 60–103. https://doi.org/10.1002/anie.200802248.

(30) Marks, L. D.; Peng, L. Nanoparticle Shape, Thermodynamics and Kinetics. *J. Phys. Condens. Matter* **2016**, *28* (5), 053001. https://doi.org/10.1088/0953-8984/28/5/053001.

(31) Thanh, N. T. K.; Maclean, N.; Mahiddine, S. Mechanisms of Nucleation and Growth of Nanoparticles in Solution. *Chem. Rev.* **2014**, *114* (15), 7610–7630. https://doi.org/10.1021/cr400544s.

(32) Galanakis, I.; Papanikolaou, N.; Dederichs, P. H. Applicability of the Broken-Bond Rule to the Surface Energy of the Fcc Metals. *Surf. Sci.* **2002**, *511* (1–3), 1–12. https://doi.org/10.1016/S0039-6028(02)01547-9.

(33) Vitos, L.; Ruban, A. V.; Skriver, H. L.; Kollár, J. The Surface Energy of Metals. *Surf. Sci.* **1998**, *411* (1–2), 186–202. https://doi.org/10.1016/S0039-6028(98)00363-X.

(34) Klinger, M. More Features, More Tools, More CrysTBox. *J. Appl. Crystallogr.* **2017**, *50* (4), 1226–1234. https://doi.org/10.1107/S1600576717006793.

(35) Jain, A.; Ong, S. P.; Hautier, G.; Chen, W.; Richards, W. D.; Dacek, S.; Cholia, S.; Gunter, D.; Skinner, D.; Ceder, G.; Persson, K. A. Commentary: The Materials Project: A Materials Genome Approach to Accelerating Materials Innovation. *APL Mater.* **2013**, *1* (1). https://doi.org/10.1063/1.4812323.

(36) Tolman, C. A. Steric Effects of Phosphorus Ligands in Organometallic Chemistry and Homogeneous Catalysis. *Chem. Rev.* **1977**, *77* (3), 313–348. https://doi.org/10.1021/cr60307a002.

(37) Kühl, O. Phosphorus-31 NMR Spectroscopy: A Concise Introduction for the Synthetic Organic and Organometallic Chemist. *Phosphorus-31 NMR Spectrosc. A Concise Introd. Synth. Org. Organomet. Chem.* **2009**, 1–131. https://doi.org/10.1007/978-3-540-79118-8.

(38) Wang, Y.; Peng, H. C.; Liu, J.; Huang, C. Z.; Xia, Y. Use of Reduction Rate as a Quantitative Knob for Controlling the Twin Structure and Shape of Palladium Nanocrystals. *Nano Lett.* **2015**, *15* (2), 1445–1450. https://doi.org/10.1021/acs.nanolett.5b00158.

(39) Nguyen, Q. N.; Chen, R.; Lyu, Z.; Xia, Y. Using Reduction Kinetics to Control and Predict the Outcome of a Colloidal Synthesis of Noble-Metal Nanocrystals. *Inorg. Chem.* **2021**, *60* (7), 4182–4197. https://doi.org/10.1021/acs.inorgchem.0c03576.

(40) Zaza, L.; Stoian, D. C.; Bussell, N.; Albertini, P. P.; Boulanger, C.; Leemans, J.; Kumar, K.; Loiudice, A.; Buonsanti, R. Increasing Precursor Reactivity Enables Continuous Tunability of Copper Nanocrystals from Single-Crystalline to Twinned and Stacking Fault-Lined. *J. Am. Chem. Soc.* **2024**, *146* (47), 32766–32776. https://doi.org/10.1021/jacs.4c12905.
